# Exploring the Antimicrobial Potential of Vanadium‐Based MXenes for Biomedical Applications

**DOI:** 10.1002/mbo3.70309

**Published:** 2026-05-18

**Authors:** Roberto Rosato, Andreas Rosenkranz, Giordano Perini, Giulia Santarelli, Alberto Augello, Dario F. Zambrano, Iasi Cervantes, Nuria Abigail Plebani, Fernando Pablo Cometto, Marco De Spirito, Maurizio Sanguinetti, Valentina Palmieri, Massimiliano Papi, Flavio De Maio

**Affiliations:** ^1^ Department of Basic Biotechnological Sciences, Intensive and Perioperative Clinics Università Cattolica del Sacro Cuore Rome Italy; ^2^ Department of Chemical Engineering, Biotechnology and Materials (FCFM) Universidad de Chile Santiago Chile; ^3^ ANID – Millennium Science Initiative Program, Millennium Nuclei of Advanced MXenes for Sustainable Applications (AMXSA) Santiago Chile; ^4^ Departamento de Fisicoquímica, Facultad de Ciencias Químicas Universidad Nacional de Córdoba Córdoba Argentina; ^5^ Department of Neuroscience Catholic University of the Sacred Heart Rome Italy; ^6^ Consejo Nacional de Investigaciones Científicas y Técnicas (CONICET), Instituto de Investigaciones en Fisicoquímica de Córdoba (INFIQC) Córdoba Argentina; ^7^ Institute for Complex Systems, National Research Council (ISC‐CNR) Rome Italy; ^8^ Department of Laboratory and Hematological Sciences Fondazione Policlinico Universitario A. Gemelli IRCCS Rome Italy

**Keywords:** 2D materials, antimicrobial activity, *Escherichia coli*, infection models, MXenes, *Staphylococcus aureus*

## Abstract

MXenes, a family of two‐dimensional transition metal carbides, carbonitrides and nitrides, have emerged as highly interesting antimicrobial nanomaterials. While mostly Ti‐based MXenes have been explored in this field, our work aims at characterizing and investigating the antibacterial and biocompatibility profiles of two vanadium‐based MXenes (V₂CT*ₓ* and V₄C₃T*ₓ*) against *Escherichia coli* and *Staphylococcus aureus*, two clinically relevant pathobionts. First, the as‐synthesized nanomaterials were chemically and structurally characterized to confirm their morphology and structural integrity. After setting up two distinct experimental models (static and dynamic), the antibacterial activity was evaluated by colony‐forming units (CFUs) counting and scansion electron microscopy (SEM). Cellular cytotoxicity was assessed by lactate dehydrogenase (LDH) release and crystal violet characterization (CV). To further evaluate the MXenes' properties, the antimicrobial activity was tested in in‐vitro infection models using both epithelial (Caco‐2) and macrophage (J774) cells measuring CFUs. To assess the oxidative stress contributing to MXenes' antibacterial activity, reactive oxygen species (ROS) production was valued in infected cells after treatment. V₂CT*ₓ* and V₄C₃T*ₓ* showed an antibacterial activity concentration and condition dependent. The dynamic incubation improved the bacterial reduction, supporting a “nano‐knife” mechanism linked to the physical disruption of the membrane. Finally, V₂CT*ₓ* and V₄C₃T*ₓ* significantly reduced the intracellular bacterial burden in infected Caco‐2 epithelial cells in comparison with macrophages. Importantly, MXenes' treatment did not result in marked ROS stimulation, suggesting that their antibacterial activity mainly arose from physical interactions. Our findings highlight that vanadium‐based MXenes have good biocompatibility and are moderately effective antimicrobial nanomaterials, emphasizing the need to use commonly recognized and standardized experimental models to elucidate their potential antimicrobial applications.

## Graphical abstract

Vanadium‐based MXenes (V₂CT*ₓ* and V₄C₃T*ₓ*) exhibit moderate antibacterial activity against *E. coli* and *S. aureus*, mainly through mechanical “nano‐knife” membrane disruption rather than ROS generation. These nanomaterials show good biocompatibility and highlight the importance of standardized experimental models for evaluating MXene antimicrobial potential.

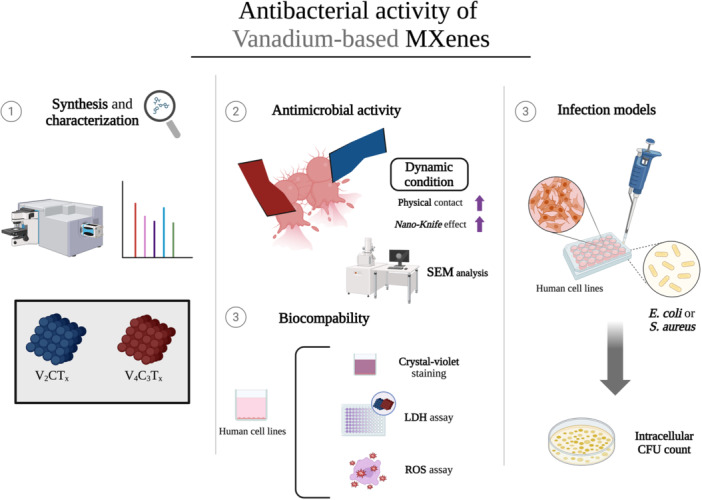

## 1 Introduction

The global rise in antimicrobial resistance has prompted the search for sustainable alternatives to the use of antibiotics in the field of nanomaterials, which possess several properties applicable in medicine and biomedical sciences (Organization, W.H 2022; Mabrouk et al. 2021; Gujjar et al. 2025). There is a multiplicity of emerging approaches, ranging from host‐directed therapies (Wallis et al. 2023; Gengenbacher et al. 2017), to innovative antimicrobial strategies based on the promising activity of nanomaterials although the lack of experimental standardization (De Maio 2019a; Fadeel et al. 2018).

The growing interest in nanomaterials relates to their adaptability due to tunable chemical and physical properties, which allow them to interfere with micro‐organisms through multiple mechanisms (Makabenta et al. 2021; Wang et al. 2017). Among the enormous plethora of nanomaterials, carbon‐based nanomaterials (CNMs), such as graphene, graphene oxide, carbon nanotubes, and quantum dots, represent one of the most promising classes due to their antimicrobial potential (De Maio 2019a; Papi et al. 2023).

Moreover, graphene quantum dots (GQDs) are capable of exhibiting a notable activity against *Escherichia coli*, particularly when combined with blue light exposure (Rosato et al. 2024). For the same material, a modest effectiveness has been observed on *Mycobacterium tuberculosis* (Mtb) (Santarelli et al. 2024). Conversely, nanomaterials having larger surface area, such as graphene oxide, have been demonstrated to inhibit Mtb entry in eukaryotic cells and induce a synergic activity when combined with linezolid (De Maio 2020, 2019b). Similarly, applications such as graphene oxide‐based coatings have shown a reduction in adhesion and biofilm formation of *Candida albicans*, while their antimicrobial activity has also been demonstrated against bacterial pathogens including *Escherichia coli* and *Staphylococcus aureus* (Palmieri et al. 2018, 2017; Papi et al. 2016).

The antimicrobial potential of nanomaterials is not driven by a single mechanism of action, but rather multiple factors. Some nanomaterials are capable of physically disrupting bacterial membranes due to their conformation, while others generate reactive oxygen species (ROS), which induce oxidative stress and damage lipids, proteins, and nucleic acids (Parvin et al. 2025; Cao et al. 2024). Furthermore, some nanomaterials exploit effects related to photothermal and photodynamic phenomena, converting light energy into heat or oxidizing radicals to enhance their antimicrobial effect (Yan et al. 2023). Furthermore, given the relevance of oxidative stress in nanomaterial–bacteria interactions with the host cells (De Maio et al. 2020), the evaluation of intracellular ROS generation is considered as a significant parameter to discriminate between physical direct antibacterial effect and host indirect cell–mediated activity. With respect to carbonaceous materials, the so‐called “trapping effect” has been further described, which consists of trapping bacteria in graphene networks, reducing their access to host cells (De Maio 2019b).

Although nanomaterials have demonstrated a notable potential, their clinical use is often disadvantaged by toxicity issues, usually related to their size, concentration, or surface functionalization (Egbuna et al. 2021; Awashra and Młynarz 2023; Salustri et al. 2023). Although higher doses can improve the antimicrobial efficacy, this approach also runs the risk of increasing the likelihood of damage to eukaryotic cells (Lalwani et al. 2016; Liang et al. 2021). Nevertheless, although toxicity represents a limitation for biomedical applications, nanomaterials can be still successfully employed in technological contexts, such as the functionalization of personal protective equipment (PPE), where they could provide additional antimicrobial and antiviral properties (Palmieri et al. 2021; De Maio et al. 2021, 2022; Maio et al. 2022).

Regarding the exploration of novel antimicrobial approaches, the research focus has recently shifted from carbon nanomaterials to MXenes, a family of two‐dimensional materials composed of transition metal carbides, nitrides, and carbonitrides. These were first synthesized in 2011 by Naguib et al (Naguib et al. 2011) and possess several unique properties, including a high surface area, hydrophilicity, an excellent electrical conductivity, and a surface chemistry that can be easily modified (Anasori et al. 2017; Bilibana 2023). These characteristics have made it possible to evaluate MXenes as promising candidates in different biomedical fields, such as biosensing, tissue regeneration, or oncology (Lin et al. 2020; Gürbüz and Ciftci 2024; Iravani and Varma 2022).

MXenes have been attributed to several mechanisms through which they can exhibit their potential antibacterial activity based on rupturing the bacterial membrane, generating ROS, and photothermal deactivating of bacteria (Rosenkranz et al. 2021; Seidi et al. 2023). Specifically, the physical disruption of bacterial membranes is facilitated by their sharp layer edges, which act as so‐called “nano‐knives,” or due to their photothermal and photodynamic effects, further promoting bacterial inhibition through localized heating and the production of singlet oxygen (Liu et al. 2023; Gnanasekar et al. 2025; Ye et al. 2024). In addition to their antibacterial effects, MXenes have also shown a good antiviral potential, as reported in a pilot study, where these nanomaterials demonstrated the capability to inhibit SARS‐CoV‐2 replication (Unal et al. 2021).

Despite some advanced applications in the biomedical field, critical issues remain that hinder their use. Differences in synthetic methodologies, non‐standardized tests in which they are used, and evaluation methods make it difficult to compare the results obtained (Akhter and Maktedar 2023; Rems et al. 2024; Liu et al. 2011). Furthermore, evaluating the safety of MXenes towards eukaryotic systems remains an area with a paucity of information on their mode of action in infection models that simulate the interaction between bacteria and host cells (Iravani and Varma 2022; Wu et al. 2023; Hansen et al. 2024).

Finally, although several studies have reported a notable antibacterial activity of Ti₃C₂T_x_ against both Gram‐negative (e.g. *E. coli* and *Pseudomonas aeruginosa*) and Gram‐positive (e.g. *Staphylococcus aureus* and *Bacillus subtilis*) bacteria, the MXene family offer many other options based on different early transition metals, which have been scarcely explored until now (Rasool et al. 2016, 2017; Santos 2022; Wu et al. 2022; Shamsabadi et al. 2018).

Compared to the widely studied Ti₃C₂T*ₓ*, vanadium‐based MXenes (V₂CT*ₓ* and V₄C₃T*ₓ*) exhibit a distinct surface chemistry and redox behavior arising from the multivalent nature of vanadium (V³⁺/V⁴⁺/V⁵⁺). These materials possess a larger active area per mass and presence of more active transition metals, which may contribute to a more pronounced antibacterial activity (Satishkumar et al. 2024). Recent studies have further highlighted that vanadium‐based MXenes possess enhanced redox and catalytic surface properties compared to other MXene compositions, which may promote stronger interactions with biological systems and improve their antimicrobial and biomedical potential (Li et al. 2024).

Following this hypothesis, our study aims at testing two vanadium‐based MXenes (V₂CT*ₓ*and V₄C₃T*ₓ*) on *E. coli* ‐based infection model. This micro‐organism represents not only a clinically relevant Gram‐negative pathogen, but also a reference intestinal bacterium that our group has already successfully employed to investigate graphene‐based nanomaterials (Rosato et al. 2024). In parallel, *Staphylococcus aureus* was included as a clinically relevant Gram‐positive opportunistic pathogen to broaden the biological relevance of the antibacterial assessment. Furthermore, intracellular ROS production was evaluated to investigate the contribution of oxidative stress to MXene–host cell interactions. Based on this rationale, our study expects to provide new insights into the antimicrobial and biocompatibility profiles of MXenes and clarify their potential role as next‐generation nanomaterials with antimicrobial activity.

## 2 Methods

### 2.1 MXenes Synthesis and Characterization

Commercial MAX‐phase precursors in powder form (V_2_AlC and V_4_AlC_3_, Forsman China) were used as starting materials for the respective selective etching treatment. To generate multi‐layer V_2_CT_x_, about 0.5 g of V_2_AlC was mixed under continuous stirring with 10 mL 28 M HF (49% HF, Sigma Aldrich). Stirring was performed under room temperature for about 2 days. In case V_4_C_3_T_x_, 0.5 g of the parental MAX phase V_4_AlC_3_ was submerged in pure HF for about 96 h at 35 C. For both procedures, de‐ionized water was subsequently added to the acidic suspension. Subsequently, several centrifugation cycles were performed at about 8000 rpm for 5 min each. After each centrifugation step, the supernatant was removed and redispersed in de‐ionized water until it reached a pH of 6. After vacuum filtration using a PVDF membrane, the respective reaction product was dried overnight in a vaccum oven to obtain the desired multi‐layer powder.

After the synthesis of V‐based MXenes (V_2_CT_x_ and V_4_C_3_T_x_) from their respective MAX precursors through selective HF etching, the powders were structurally and morphologically characterized. The morphology and chemical composition of both MXenes were assessed by field‐emission scanning electron microscopy combined with energy‐dispersive X‐ray spectroscopy (FE‐SEM/EDS, Inspect F50, FEI) and confocal Raman spectroscopy. Raman spectra were acquired using a LabRAM HR Evolution spectrometer (Horiba) in backscattering geometry with a 532 nm laser excitation source. The nominal laser power of 105 mW was attenuated to approximately 2 mW to minimize local heating and structural degradation. All spectra were recorded in the 80–1000 cm^−1^ range with a total integration time of 128 s per scan. A diffraction grating of 1800 lines/mm provided a spectral resolution of about 3 cm^‐1^. Finally, the crystalline structure was studied by X‐ray diffraction (XRD) using a Bruker D8 Advance diffractometer equipped with Cu Kα radiation (*λ* = 1.5406 Å). The corresponding diffractograms were collected within a 2θ range of 5°–65°, with a step size of 0.02° s^−1^, employing the conventional θ–2θ Bragg‐Brentano configuration.

### 2.2 X‐Ray Photoelectron Spectroscopy (XPS) Analysis

XPS analyses were performed using a Thermo Scientific K‐Alpha+ spectrometer. Measurements were carried out at room temperature under ultra‐high vacuum conditions (UHV ≈ 1 × 10^−6^ Pa). A non‐monochromatic Al Kα X‐ray source (1200 W) was employed for sample excitation, and photoelectrons were collected using a 180° double‐focusing hemispherical analyzer from an analysis area of approximately 200–300 μm in diameter. Spectra were acquired in normal emission geometry, corresponding to a 90° take‐off angle relative to the sample surface. Survey (wide‐scan), valence band (VB), and high‐resolution spectra of the C 1 s, O 1 s, F 1 s, and V 2p core levels were recorded. The pass energy and step size parameters were set to 100 eV/0.5 eV for survey spectra, 50 eV/0.05 eV for VB spectra, and 20 eV/0.02 eV for high‐resolution spectra, respectively. Sputter‐cleaning was performed using an Ar⁺ ion beam, calibrated to achieve a material removal rate of approximately 1 nm s^−1^. Spectral deconvolution was carried out using Avantage v5.9912 and OriginPro 2025b software, employing Voigt line shapes. Binding energy (BE) calibration followed the procedure proposed by Näslund et al (Näslund and Persson 2022)., wherein each high‐resolution spectrum was corrected by the energy shift of the Fermi edge (Figure S1a) observed in the VB spectrum with respect to BE = 0.0 eV. This shift was determined by calculating the first derivative of the VB spectrum around the Fermi level (gray curve in Figure S1b). The resulting correction of approximately 0.35 eV was applied to all VB and core‐level spectra (C 1 s, O 1 s, V 2p, and F 1 s) for each sample analyzed.

### 2.3 Sample Preparation for XPS Analysis

Aqueous dispersions of V_4_C_3_T_*x*_ and V2CT*x* MXenes were prepared using milli‐Q water to obtain a concentrated stock dispersion, which was subsequently diluted in a 2:1 ratio. For each MXene, four types of samples were prepared by the drop‐casting method on gold substrates to assess the influence of different treatments on surface chemistry: fresh—40 µL of freshly prepared dispersion deposited on an Au substrate; aged—40 µL of dispersion was deposited on an Au substrate 6 days after preparation; ann—aged sample annealed with a butane flame at 500°C and 1000°C for a few seconds after water evaporation; sputt ‐ aged sample sputter‐cleaned for 5 s by using an Ag+ ion beam.

Gold substrates consisted of evaporated Au films on flat glass (Arrandee). Prior to MXene deposition, the substrates were annealed using a butane flame (two cycles of 2 min each, in the dark) to remove residual organic contaminants from the surface.

### 2.4 Bacterial Manipulation

*Escherichia coli* and *Staphylococcus aureus* were grown in a Luria‐Bertani (LB) broth medium (Sigma‐Aldrich, Saint Louis, MO, USA) at 37°C overnight. The bacterial cultures were then harvested, and 20% sterile pure glycerol (Carlo Erba Reagents, Milano, Italy) was added. Aliquots of each suspension were finally stored at −80°C and then assessed in terms of colony‐forming units (CFUs). All experiments that involved both *E. coli* and *S. aureus* manipulation were performed in Biosafety level 2 laboratory in the Institute of Microbiology of Fondazione Policlinico Universitario A. Gemelli‐IRCCS.

### 2.5 In vitro Antimicrobial Assay

To evaluate the in vitro antimicrobial activity of V_2_CT_x_ and V_4_C_3_T_x_ against *E. coli* and *S. aureus*, a 96‐well plate (Corning, New York, NY, USA) was prepared with suspensions of each MXene at final concentrations of 500, 200, 100, and 50 µg/mL, respectively. The bacterial suspensions were added to each well to achieve a final bacterial concentration of 5 × 10^5^ CFU/mL as previously reported (Rosato et al. 2024; Santarelli et al. 2024). The 96‐well plate was then incubated at 37°C for two different time points of 4 and 24 h.

Concurrently, 3 mL suspensions were prepared in 8 mL sterile tubes (Fisher Scientific, New Hampshire, USA) using the previously mentioned concentrations and incubated at 37°C on a rotating wheel for 4 h and 24 h, respectively to simulate dynamic conditions.

For each designated time point, CFUs count was performed to quantify bacterial viability. Positive (*E. coli* or *S. aureus* without MXenes) and negative (LB broth medium only) controls were included in all experiments.

### 2.6 Sample Preparation for Scanning Electron Microscopy (SEM) Analysis

For scanning electron microscopy (SEM) analysis, treated and untreated microbial samples were prepared as follows. Briefly, bacterial suspensions of *E. coli* or *S. aureus*, at a final concentration of 5 × 10^5^ were incubated for 4 h at 37°C under the experimental conditions described above, including untreated controls and treatment with V₂CTₓ or V₄C₃Tₓ MXenes under static or dynamic incubation conditions. The following experimental groups were included: untreated bacteria (positive infection control), kanamycin‐treated bacteria at its respective minimum inhibitory concentration (MIC) for each strain (negative control), and bacteria exposed to V₂CTₓ or V₄C₃Tₓ MXenes, each tested at a single concentration of 500 µg/mL under either static or dynamic incubation.

After incubation, the content of each 1.5 mL tube was collected, and 50 µL of the diluted suspension was deposited onto transparent plastic disks (NUNC Brand Products, Rochester, NY, USA), which were placed in an incubator at 37°C until complete dehydration.

Samples were then fixed by immersion in 2.5% (v/v) glutaraldehyde solution for 15 min at room temperature. Following fixation, disks were washed twice with 400 µL of distilled water to remove residual fixative. Dehydration was performed through a graded ethanol series, consisting of sequential incubations in 30%, 50%, 70%, 90%, and 100% ethanol solutions for 10 min each. After completion of the dehydration steps, samples were allowed to air‐dry to ensure complete evaporation of residual ethanol.

The dried disks were mounted onto aluminum SEM stubs using conductive carbon adhesive tabs and sputter‐coated with a 15 nm platinum layer using a high‐resolution sputter coater (AGB7234, UK), operated at a current of 40 mA. SEM images were acquired using a Zeiss Supra 25 scanning electron microscope (Zeiss, Germany) at different magnifications ranging from 1.15k× to 6.52k×. Representative micrographs were selected from at least three independent experiments.

### 2.7 Evaluation of Cytotoxicity on Eukaryotic Cells

To assess the cell viability following the treatment with MXenes, human colorectal adenocarcinoma cells (Caco‐2 cell line) were cultured and subsequently seeded at a final concentration of 5 × 10^5^ cells/mL in 96‐well plates (Corning, New York, NY, USA). Caco‐2 cells were incubated overnight under standard atmospheric conditions (37°C and 5% CO₂), followed by treatment with either V_2_CT_*x*_ and V_4_C_3_T_*x*_ at final concentrations of 500, 200, 100 and 50 µg/mL, respectively. Untreated cells served as the negative control, while cells exposed to 2% Triton X‐100 (Sigma‐Aldrich, USA) were used as the positive control. Triton X‐100 is known to disrupt cellular membranes by solubilizing the lipid bilayer, resulting in complete cell lysis (Koley and Bard 2010; Mattei et al. 2017). Cells were incubated for 24 h, after which lactate dehydrogenase (LDH) release was measured to assess cytotoxicity (Salustri et al. 2023).

Briefly, LDH activity was quantified in cell culture supernatants, which were centrifuged to remove residual MXenes. Supernatants were diluted accordingly and incubated with the substrate solution. After 30 min, absorbance at 490 nm and 680 nm was recorded using an automatic microplate reader (Cytation 5 Cell Imaging Multi‐Mode Reader, Biotek Instruments, USA). The percentage of LDH‐mediated cytotoxicity was calculated using the following equation: % Cytotoxicity = [(Compound LDH activity – Spontaneous LDH activity)/(Maximum LDH activity – Spontaneous LDH activity)] × 100, where Spontaneous LDH activity corresponds to untreated cells and Maximum LDH activity to cells treated with Triton X‐100. To strengthen the biocompatibility assessment, we carried out crystal violet (CV) staining of the MXenes‐treated cell monolayer as described above (De Maio et al. 2021). Briefly, CV labels the DNA of live, adherent cells and was used to quantify viable cells. Cells were plated as described above and then stained by using CV for 30 min. After incubation, five washes were performed, and images of random fields for each condition were acquired (De Maio et al. 2021).

### 2.8 Human Colorectal Adenocarcinoma Cells Culture and Infection

Caco‐2 cells (ATCC, Manassas, VA, USA; Accession Number: CVCL_0025) were cultured in DMEM (Euroclone, Milano, Italy) supplemented with 20% inactivated FBS (Euroclone, Milano, Italy), 1% L‐glutamine (Euroclone, Milano, Italy), and 1% streptomycin–penicillin (Euroclone, Milano, Italy), and incubated at 37°C in a humidified atmosphere with 5% CO₂. Adherent cells were washed with sterile warm PBS (Euroclone, Milano, Italy) and detached using 1× trypsin in PBS (Euroclone, Milano, Italy) for subsequent experiments. Cells were counted and re‐suspended in DMEM supplemented with 2% FBS and 1% L‐glutamine. Finally, cells were seeded in sterile 48‐well plates (Euroclone, Milano, Italy) at a concentration of 5 × 10⁵ cells/mL and incubated overnight prior to infection or treatment.

Caco‐2 cells were infected with *E. coli* or *S. aureus* at a multiplicity of infection (MOI) of 100 (100 bacteria per cell), with bacteria resuspended in cell culture medium. 2‐h post‐infection, cells were washed with sterile warm PBS to remove extracellular bacteria and subsequently treated with either V_2_CT_x_ and V_4_C_3_T_x_ suspensions at final concentrations of 500, 200, 100 and 50 µg/mL, respectively. Following the treatment, cells were incubated under standard conditions for 4 and 24 h.

To assess intracellular bacterial survival, the cell monolayer was harvested using 0.1 mL of sterile 0.05% Triton X‐100 (Sigma‐Aldrich, USA). Serial 1:10 dilutions of the lysates were plated on LB agar plates and incubated overnight at 37°C. CFUs were calculated by multiplying the number of colonies by the corresponding dilution factor and reported as log intracellular CFU. The schematic flowchart is reported in results.

### 2.9 Evaluation of Intracellular ROS Production

To evaluate intracellular ROS production following MXene treatment, Caco‐2 cells were infected with *E. coli* or *S. aureus* at a multiplicity of infection (MOI) of 100 for 2 h. After removal of extracellular bacteria and washing with PBS, cells were treated with V_2_CT_*x*_ and V_4_C_3_T_x_ at final concentrations of 500, 200, 100 and 50 µg/mL or maintained in fresh culture medium. In parallel, uninfected Caco‐2 cells were exposed to equivalent concentrations of V_2_CT_*x*_ and V_4_C_3_T_x_ to evaluate ROS generation directly attributable to MXene treatment in the absence of bacterial challenge.

For both 4 and 24 h time points, cells were incubated with 2′,7′‐dichlorofluorescein diacetate (DCFDA; Sigma‐Aldrich, USA) according to the manufacturer's instructions. Fluorescence intensity was measured using a Cytation 5 Cell Imaging Multi‐Mode Reader (Biotek Instruments, USA), and ROS levels were expressed as percentage relative to untreated‐uninfected controls. Tert‐butyl hydroperoxide (TBHP) was used as a positive control for ROS induction.

### 2.10 Statistical Analysis

Data were collected and organized using Microsoft Excel software version 16.89.1 and were analyzed by using GraphPad Prism software version 10.4.2 (GraphPad software) and R v4.5.0. All experiments were performed in scientific duplicates and technical triplicates. All data were expressed as mean plus SD and analyzed by two‐way ANOVA followed by the appropriate correction.

## 3 Results

### 3.1 Structural, Morphological and Chemical of V_2_CT_x_ and V_4_C_3_T_x_

**Figure 1:**
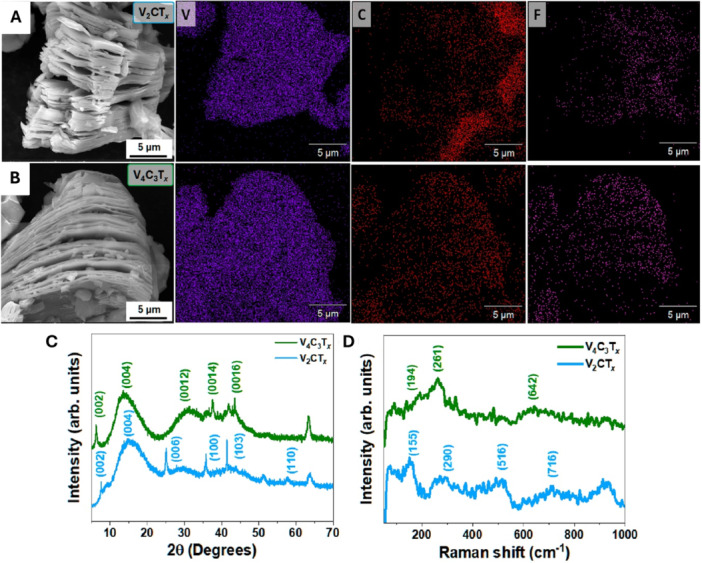
V2CTx and V4C3Tx MXenes show distinct morphological and structural features by SEM, XRD and Raman analysis. The distinctive characteristics of V₂CT*x* and V₄C₃T_x_ can be observed in the micrographs acquired using SEM microscopy (A, B). V₂CT*x* exhibits a morphology characterized by thin sheets, irregular stacking, and folded edges, whereas V₄C₃T_x_ is characterized by thicker, more compact sheets. A homogeneous distribution of vanadium and carbon can be observed through elemental EDS mapping, which also shows signals of oxygen and fluorine associated with surface terminations. A dominant peak (002) is visible in the XRD analysis (C) in both MXenes, where V₂CT*x* shows additional reflections that are related to its structure, and higher‐order peaks in V₄C₃T_x_ that suggest its multilayer periodicity. The two MXenes were clearly distinguished by Raman spectroscopy (D), revealing characteristic peaks for V₂CT*x* and a more complex vibrational profile for V₄C₃T*x*, which aligns with its greater structural complexity.

The morphological characterization of vanadium‐based MXenes by SEM reveals distinct features for V_2_CT_x_ and V_4_C_3_T_x_. In the SEM micrograph of V_2_CT_*x*_ (Figure 1a), the nano‐sheets exhibit a lamellar morphology with thin sheets, irregular stacking, and folded edges, indicative for an effective transformation of the parental MAX‐phase into the targeted V_2_CT_x_ MX. The elemental EDS mapping confirms a homogeneous distribution of vanadium and carbon, which resembles the main MXene elements as well as fluorine and oxygen, which can be associated with surface terminations. These results validate the chemical uniformity of the material after HF etching (Chhattal and Grützmacher 2024). In contrast, V_4_C_3_T_x_ (Figure 1b) displays thicker and more compact sheets, consistent with its higher structural complexity derived from the V_4_AlC_3_ MAX phase. The chemical mapping again shows a homogeneous distribution of V and C, with O and F arising from surface terminations introduced during synthesis (Bin et al. 2022; Abraham et al. 2025).

The structural analysis by XRD (Figure 1c) demonstrates that V_2_CT_*x*_ exhibits a dominant (002) peak located at lower diffraction angles, indicating an increased interlayer spacing (~1.16 nm) after Al removal and the incorporation of ‐O, ‐OH, and ‐F terminations. Additional (004), (006), (100), (103), and (110) peaks can also be observed, which agree with its reported hexagonal structure (Chhattal and Grützmacher 2024). For V_4_C_3_T_x_, the diffractogram displays a strong (002) peak but is accompanied by a larger number of higher‐order peaks [(004), (0012), (0014), (0016)], reflecting its multilayer periodicity and greater structural stability compared to V_2_CT_x_ (Bin et al. 2022).

Raman spectroscopy (Figure 1d) clearly distinguishes between both MX structures. For V_2_CT_x_, characteristic peaks appear at 379, 495, and 630 cm⁻¹, corresponding to A1g (out‐of‐plane) and Eg (in‐plane) vibrational modes of vanadium, together with secondary bands near 800 cm^−1^ associated with ‐F/‐OH terminations (Chhattal and Grützmacher 2024). In contrast, V_4_C_3_T_x_ exhibits a more complex spectrum, with well‐defined peaks at 194, 261, and 642 cm^−1^, consistent with its multilayer structure and the larger number of Raman‐active modes since the number of layers per unit cell increases (Shevchuk et al. 2023).

### 3.2 X‐Ray Photoelectron Spectroscopy (XPS) Analysis

**Figure 2:**
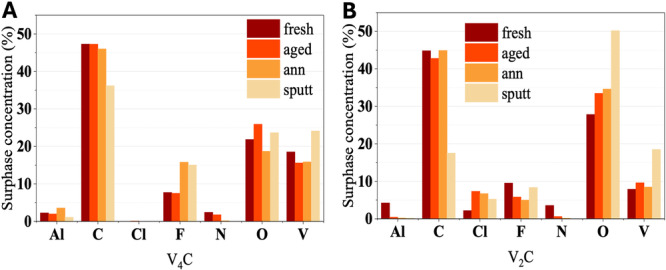
Elemental composition of (A) V₄C₃Tx and (B) V₂CTx MXene samples under different conditions, expressed as atomic percentages derived from XPS survey spectra.

**Figure 3:**
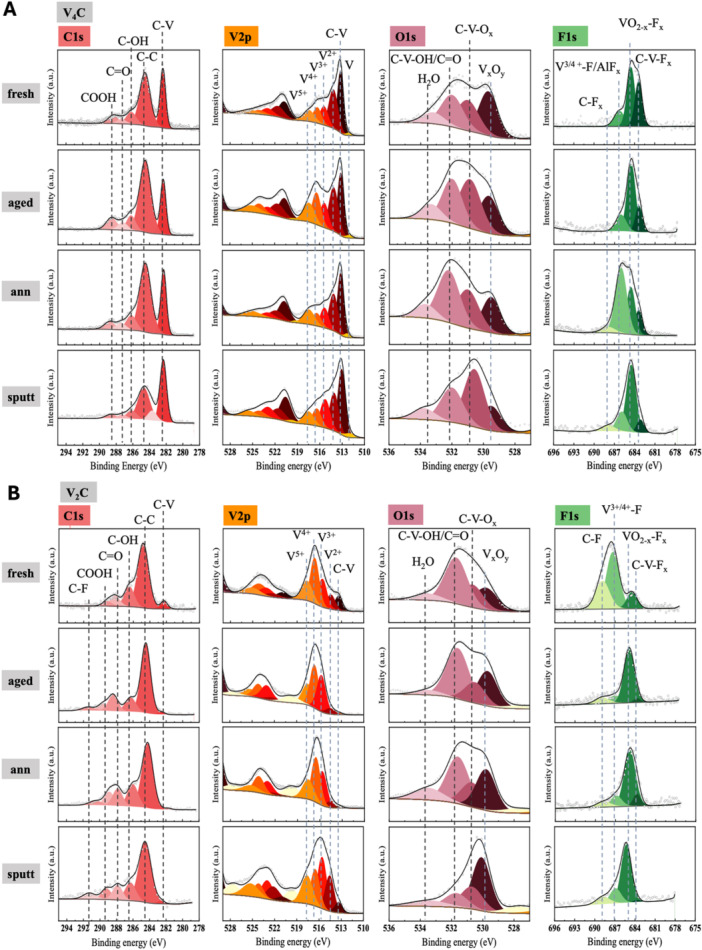
High‐resolution XPS spectra of the C 1 s, V 2p, O 1 s, and F 1 s core levels for (A) V₄C₃Tx and (B) V₂CTx MXene samples prepared under different conditions.

To investigate the elemental composition and the chemical environment of the V_4_C_3_T_x_ and V_2_CT_x_ samples, a series of X‐ray photoelectron spectroscopy (XPS) measurements was performed. From the survey spectra, the atomic percentages of each element present under the four different conditions (fresh, aged, annealed, and sputtered) were determined for both MXenes (Figure 2). As shown, significant compositional changes occur depending on the treatment applied. For the V_4_C_3_T_x_ sample (Figure 2a), storage in Milli‐Q water for 6 days led to an increase in oxygen content, accompanied by a moderate decrease in vanadium. This variation can be attributed to oxidation in aqueous environments, where water and dissolved oxygen promote the formation of surface superficial oxide species and modifications of the surface terminations. Notably, as widely reported, XPS probes only the outermost layers; therefore, the observed oxidation is predominantly a surface phenomenon and does not necessarily imply a complete structural degradation of the MXene. Upon thermal annealing, a pronounced increase in surface fluorine content was observed compared to the fresh and aged samples, suggesting the formation or segregation of F‐containing surface species. In contrast, the same treatment applied to the V_2_CT_*x*_ sample (Figure 2b) did not induce comparable changes: Although an increase in oxygen content is observed, vanadium shows a similar trend, which is accompanied by a marked decrease in aluminum, nitrogen, and fluorine. Finally, for the sputtered surfaces of both MXenes, a clear increase in the relative amounts of V, F, and O was detected with respect to the untreated surfaces. This behavior can be ascribed to the removal of adventitious carbon and organic contaminants, yielding a surface enriched in oxygenated and fluorinated species. The high‐resolution C1s spectra of both MXenes, V_4_C_3_T_x_ (Figure 3a) and V_2_CT_*x*_ (Figure 3b) summarized in Tables 1 and 2, reveal three main carbon types. The first corresponds to C─V bonds at 282.3 eV, characteristic of the MXene structure (Wu et al. 2024; Wang et al. 2025; Du et al. 2020; Feng et al. 2024). A prominent peak at approximately 285 eV is assigned to C–C bonds in amorphous or graphitic carbon (Wu et al. 2024; Wang et al. 2025; Du et al. 2020; Feng et al. 2024; Blyth et al. 2000) commonly formed during the etching and delamination of the MAX phase precursor (Lukatskaya et al. 2014). Additional contributions are observed at higher binding energies, corresponding to C─H (~285.0 eV), C─OH (~286.3 eV), C═O (287.2–287.6 eV), COOH (288.5–289.0 eV), CO₃²⁻ (289.0–290.0 eV), and C─F (~291.7 eV) species (Wang et al. 2025; Blyth et al. 2000) (Henderson et al. 2025; Schier and Halbritter 1993; Sultana et al. 2008). These components likely originate from residual solvents used during washing or dispersion, as well as from the exposure of highly reactive MXene surfaces to the ambient atmosphere. The O1s spectra of V_4_C_3_T_x_ (Figure 3a) and V_2_CT_x_ (Figure 3b) consist of four main components: V*ₓ*Oγ (~529.6 eV), C–V–O_*x*_ (~530.7 eV), C–V–OH/RO (531.6–532.1 eV), and adsorbed H₂O, respectively (Wu et al. 2024; Wang et al. 2025; Du et al. 2020). The latter originates from both the MXene synthesis and dispersion steps, since Milli‐Q water was used throughout. In the V_4_C_3_T_*x*_ fresh sample (Figure 3a and Table 1), the spectrum is dominated by the C–V–O_*x*_ and V_x_O_γ_ components, whereas in V_2_CT_x_ fresh (Figure 3b and Table 2), the main contribution arises from C–V–OH and organic species such as C=O, indicating greater reactivity toward atmospheric oxygen and moisture. After several days of storage in water, both samples show an increase in C–V–OH and V_x_O_γ_ species, highlighting their susceptibility to oxidation under ambient conditions. Upon annealing, the spectra reveal an enhancement in V–OH terminations and oxygenated organic species, more pronounced in V_4_C_3_T_x_ than in V_2_CT_x_. After ion sputtering, a marked reduction in organic components is observed, yielding surfaces enriched in V–O_x_ and vanadium oxide species. The F1s spectra of V_4_C_3_T_x_ (Figure 3a) and V_2_CT_x_ (Figure 3b) can be deconvoluted into four main components: C–V–Fₓ (683.3–683.8 eV), C–VO_2‐x_F_x_ (684.5–684.9 eV), C–V^3+^/V^4+^–F or AlF_x_ (686.2–686.9 eV), and C–F (687.9–688.8 eV), respectivley (Feng et al. 2024). The relatively low fluorine content (≤ 5 at%) suggests that F‐containing species are mainly by‐products of the MXene synthesis. In V_4_C_3_T_x_ (Figure 3a), no significant variations are detected among most conditions, except for the annealed sample, where the intensity of the C–V^3+^/^4+^–F/AlF_x_ component markedly increases, consistent with the higher overall F content observed in the survey spectra (Figure 2). Since the increase in Al is minimal (≤ 5 at%), the feature near 686.4 eV cannot be solely attributed to AlF_x_. This behavior is not observed in V_2_CT_x_, whose F1s spectra remain nearly unchanged between the fresh and aged samples. The V2p spectra of V_4_C_3_T_x_ (Figure 3a and Table 1) and V_2_CT_x_ (Figure 3b and Table 2) can be deconvoluted into six main components assigned to metallic V (~512.1 eV), C─V (~513.3 eV), V^2+^ (~514.5 eV), V^3+^ (~515.6 eV), V^4+^ (~516.6 eV), and V^5+^ (~517.6 eV), respectively. In V_2_CT_x_, even the freshly prepared sample exhibits only a weak C─V contribution, which becomes nearly negligible after several days of storage. This decreases, also evident in the C1s spectra, underscores a strong tendency of V2CTx to oxidize in aqueous environments, particularly in suspension, where hydrolysis and dissolved oxygen accelerate surface oxidation processes. Simple drying of the drop‐cast suspension on a clean Au substrate is sufficient to promote oxidation, leading to the predominance of V^5+^, V^4+^, and V^3+^ species. Flame annealing produces no significant change in oxidation‐state distribution, in agreement with the F 1 s results. After sputtering, partial reduction occurs, increasing the relative intensities of V–C, V^2+^, and V^3+^ species. In contrast, the V_4_C_3_T_x_ sample initially exhibits a strong C–V contribution and lower amounts of oxidized species (V^3+^–V^5+^). After 6 days in water, the surface becomes partially oxidized, reducing the C─V signal, although to a lesser extent than in V_2_CT_x_, indicating higher stability toward oxidation. This behavior is consistent with previously reported oxidation of MXenes in suspension, which is typically surface‐limited as detected by XPS (Habib et al. 2019; Zhang et al. 2017). In the annealed sample, a notable decrease in C–V–F and V–O_2‐x_–F_x_ terminations is accompanied by an increase in more oxidized species such as C–V^3+^/^4+^–F, consistent with the F 1s findings. Finally, after sputtering, both MXenes exhibit similar trends: reduction of oxidized species and a marked increase in the relative intensity of C─V and other low‐valence vanadium states. It is important to note that sputtering during few seconds at low energy emission does not create new C─V bonds in either material. The apparent increase in their intensity instead arises from the removal of oxidized carbon species (C─F, COOH, C═O, and C─OH) that obscure the underlying MXene signal. In summary, the XPS analysis of V_2_CT_x_ and V_4_C_3_T_x_ under different conditions allows several general conclusions to be drawn. Both samples exhibit clear C─V bonding, confirming successful MXene formation, with a higher C─V contribution in V_4_C_3_T_x_. Storage in Milli‐Q water for 6 days induces oxidation in both materials, more pronounced for V_2_CT_x_, highlighting its greater sensitivity to oxygen and aqueous environments. Flame annealing at 500°C–1000°C promotes the formation of fluorinated species in V_4_C_3_T_x_, while producing negligible changes in V_2_CT_x_. Finally, ion sputtering induces surface reduction, an effect that should be carefully considered when performing depth‐profiling analyses. MXenes dispersed in aqueous media are known to undergo progressive surface oxidation due to hydrolysis and dissolved oxygen. In this regard, the changes observed in aged samples are consistent with surface‐limited oxidation processes under biologically relevant conditions.

**Table 1:** XPS fitting parameters for V_4_C_3_Tx MXene under different surface conditions.

Region	BE [eV]	FWHM [eV]	Sample condition (fraction)				Assigned to	Reference
			*fresh*	*aged*	*ann*	*sputt*		
V2p₃/₂ (2p₁/₂)	512.1 (519.9)	1.0 (1.0)	0.01	0.01	0.02	0.03	V	
	513.1 (520.7)	1.0 (1.8)	0.33	0.28	0.36	0.36	C─V	Bin et al. (2022); Abraham et al. (2025); Shevchuk et al. (2023)
	514.1 (521.6)	1.4 (2.2)	0.30	0.26	0.26	0.22	V²⁺	Bin et al. (2022); Shevchuk et al. (2023)
	515.3 (523.4)	1.3 (2.0)	0.14	0.13	0.13	0.19	V³⁺	Bin et al. (2022); Abraham et al. (2025)
	516.4 (524.1)	1.4 (1.8)	0.10	0.17	0.08	0.10	V⁴⁺	Bin et al. (2022); Abraham et al. (2025)
	517.4 (525.5)	1.7 (2.8)	0.11	0.15	0.15	0.10	V⁵⁺	Bin et al. (2022)
C1s	282.3	1.0	0.29	0.21	0.25	0.39	C─V	Bin et al. (2022); Abraham et al. (2025); Shevchuk et al. (2023); Wu et al. (2024)
	284.7	1.6	0.50	0.56	0.55	0.44	C–C/C–H	Bin et al. (2022); Z (2025)
	286.4	1.4	0.09	0.09	0.10	0.08	C–OH/C–O–C	Z (2025); Feng et al. (2024)
	287.6	1.4	0.05	0.05	0.39	0.04	C═O	Bin et al. (2022); Feng et al. (2024)
	288.8	1.4	0.07	0.08	0.06	0.04	COOH/COOR	Z (2025); Feng et al. (2024); Blyth et al. (2000)
O1s	529.6	1.5	0.26	0.26	0.22	0.18	VₓOᵧ	Bin et al. (2022); Abraham et al. (2025)
	530.7	1.5	0.21	0.34	0.15	0.45	C–V–Oₓ	Bin et al. (2022)
	532.1	1.5	0.39	0.30	0.49	0.28	C–V–OH/C═O	Bin et al. (2022)
	533.3	1.7	0.13	0.10	0.13	0.09	H₂O	Abraham et al. (2025)
F1s	683.3	1.3	0.26	0.21	0.11	0.09	C–V–Fₓ	Wu et al. (2024)
	684.5	1.5	0.44	0.61	0.33	0.62	VO₂₋ₓ–Fₓ	
	686.2	2.0	0.30	0.19	0.50	0.20	V³⁺/⁴⁺–F	
	687.9	2.5	0.00	0.00	0.51	0.08	C–F	Wu et al. (2024)

**Table 2:** XPS fitting parameters for V₂CTx MXene under different surface conditions.

Region	BE [eV]	FWHM [eV]	Sample condition (fraction)				Assigned to	Reference
			*fresh*	*aged*	*ann*	*sputt*		
**V2p₃/₂ (2p₁/₂)**	513.3 (521.0)	1.2 (1.8)	0.11	0.01	0.01	0.05	C─V	Bin et al. (2022); Abraham et al. (2025); Shevchuk et al. (2023)
	514.5 (522.2)	1.4 (2.2)	0.11	0.06	0.06	0.22	V²⁺	Bin et al. (2022); Shevchuk et al. (2023)
	515.6 (523.1)	1.4 (1.7)	0.22	0.33	0.32	0.30	V³⁺	Bin et al. (2022); Abraham et al. (2025)
	516.6 (524.1)	1.4 (1.6)	0.36	0.35	0.39	0.20	V⁴⁺	Bin et al. (2022); Abraham et al. (2025)
	517.6 (525.1)	1.8 (2.6)	0.20	0.24	0.22	0.23	V⁵⁺	Bin et al. (2022)
**C1s**	282.6	1.0	0.05	0.01	0.01	0.01	C–V	Bin et al. (2022); Abraham et al. (2025); Shevchuk et al. (2023); Wu et al. (2024)
	284.5	1.6	0.64	0.61	0.54	0.59	C–C/C–H	Bin et al. (2022); Z (2025)
	286.3	1.5	0.18	0.13	0.17	0.15	C–OH/C–O–C	Z (2025); Feng et al. (2024)
	288.5	1.4	0.09	0.14	0.11	0.11	COOH/COOR	Z (2025); Feng et al. (2024)
	289.6	1.4	0.04	0.06	0.12	0.08	CO₃²⁻	Z (2025); Feng et al. (2024); Blyth et al. (2000)
	291.7	1.8	0	0.04	0.05	0.06	C–F	Lukatskaya et al. (2014)
**O1s**	529.8	1.6	0.23	0.29	0.30	0.49	VₓOᵧ	Bin et al. (2022); Abraham et al. (2025)
	530.6	1.5	0.21	0.18	0.19	0.24	C–V–Oₓ	Bin et al. (2022)
	531.6	1.8	0.48	0.48	0.38	0.16	C–V–OH/C = O	Bin et al. (2022)
	533.8	2.0	0.08	0.05	0.13	0.11	H₂O	Abraham et al. (2025)
**F1s**	683.8	1.2	0	0.02	0.10	0.02	C–V–Fₓ	Wu et al. (2024)
	684.9	1.8	0.13	0.74	0.69	0.74	VO₂₋ₓ–Fₓ	
	686.9	2.2	0.56	0.12	0.13	0.13	V³⁺/⁴⁺–F	
	688.8	2.5	0.31	0.11	0.08	0.10	C–F	Wu et al. (2024)

### 3.3 Concentration‐ and Dynamic‐Dependent Antibacterial Activity of V_2_CT_x_ and V_4_C_3_T_x_ MXenes

**Figure 4:**
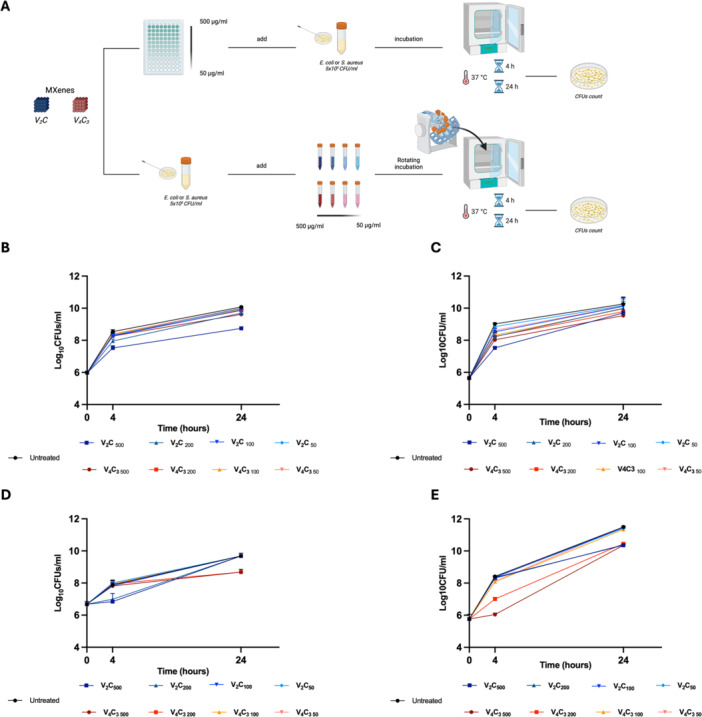
Dynamic antimicrobial properties of V₂CTx and V₄C₃Tx MXenes depend on their concentration. Schematic representation of the experimental models used to assess V₂CTx and V₄C₃Tx MXenes activity against *E. coli*. (A). A suspension of *E. coli* (5×10^5^ CFUs/ml) was incubated with V₂CTx and V₄C₃Tx MXenes at the final concentrations of 500, 200, 100 and 50 µg/mL. Colony‐forming units (CFUs) were measured at 4‐ and 24‐ hours post static (B) or dynamic (C) incubation (rotating‐incubation). Line plots described CFUs/ml, reported in log10 scale, of the initial bacterial solution and at the indicated time points. The antibacterial activity of V₂CTx and V₄C₃Tx MXenes were measured as the percentage to reduce *E. coli* growth at 4 h (D and E, static and dynamic assay, respectively). Data were expressed as mean ± SD and statistical significance assessed by one‐way ANOVA.

To assess the antimicrobial properties of both MXenes, we set up an axenic culture‐based model where *E. coli* and *S. aureus* were incubated with the nanomaterials as described above (Ye et al. 2024; Wang et al. 2025; G). The antibacterial activity of V_2_CT_x_ and V_4_C_3_T_x_ MX against the pathogens was evaluated by preparing solutions of both nanomaterials at concentrations of 500, 200, 100 and 50 µg/ml, which were then incubated with a suspension of *E. coli* or *S. aureus* at a final concentration of 5 × 10⁵ CFU/ml in a 96‐well plate. The plate was then incubated to both static and rotating culture conditions for 4‐ and 24‐h prior to assessing the antibacterial activity by counting CFUs following serial dilutions of the suspension (Figure 4A).

The CFU count showed that MX used under static conditions were able to reduce the bacterial burden of *E. coli* only at the highest concentrations of V_2_CT_x_ MX (500, 200 µg/mL) thus resulting in a slight reduction of *E. coli* (by 1 log and 0.6 log, respectively (*p* < 0.0001)) after 4 h of incubation (Figure 4B). After 24 h of incubation, the antibacterial activity remained fairly stable with an average reduction in CFUs count of 1.3 and 0.4 log for 500 and 200 µg/ml V_2_CT_x_ MX (*p* < 0.0001), respectively (Figure 4B). Comparing the percentage of reduction with respect to the control, following the treatment with V_2_CT_x_ MX, a significant decrease of the bacteria burden at concentrations of 500 µg/ml (−11.3%, *p* < 0.0001), 200 µg/mL (−6.7%, *p* < 0.0001), and 100 µg/mL (−3.1%, *p* < 0.05) was observed after 4 h of incubation (Figure S2A). Conversely, V_4_C_3_T_x_ MX was able to reduce bacterial burden only at the highest concentration (−3.2%, *p* < 0.05) (Figure S2B). For *S. aureus*, after 4 h of static incubation V₂CTₓ decreased the bacterial burden only at the highest concentrations, resulting in a reduction of 1.05 and 0.90 log for 500 and 200 µg/mL, respectively (*p* < 0.0001) (Figure 4D). When expressed as percentage variation relative to untreated controls, these reductions corresponded to −11.9% and −6.8% at 500 and 200 µg/mL, respectively (*p* < 0.0001) (Figure S2C). In contrast, V₄C₃Tₓ exerted only a minimal effect under the same conditions, with a slight reduction of 0.07 log at 500 µg/ml (*p* < 0.05), whereas 200 µg/mL and lower concentrations did not significantly influence bacterial growth (*p* > 0.05) (Figure 4D). In terms of percentage, we observed a modest −3.3% decrease at 500 µg/mL, while percentage variations at 200, 100 and 50 µg/mL were limited to −2.6%, −2.0% and −1.2%, respectively (Figure S2C). After 24 h of incubation the same conditions, the antibacterial activity was partially attenuated. Indeed, V₂CTₓ no longer displayed sustained activity compared to untreated controls (*p* > 0.05). Conversely, V₄C₃Tₓ at 500 and 200 µg/mL maintained an average reduction of approximately 1.00 log (*p* < 0.05) (Figure 4D).

To maximize the potential *“nano‐knives”* effect to physically disrupt the bacterial membrane (Gnanasekar et al. 2025; Ferrara et al. 2024), we applied rotation to the suspension of bacteria and nanomaterials. Interestingly, V_2_CT_x_ and V_4_C_3_T_x_ MX exhibited a slightly better antimicrobial property against *E. coli* under these dynamic conditions. Specifically, an average decrease in the microbial burden was measured after 4 h of incubation and it reached 1.5, 0.8 and 0.5 logs less for concentrations of 500, 200 and 100 µg/mL V_2_CT_x_ MX (*p* < 0.0001), respectively, compared to untreated bacteria. In contrast, an average decrease of approximately 1, 0.8 and 0.7 was observed when using 500, 200 and 100 µg/mL of V_4_C_3_T_x_ MX (*p* < 0.0001), respectively.

The usage of a rotating incubation has been valuable to improve the antibacterial effect of V_2_CT_x_ and V_4_C_3_T_x_ MX. Indeed, the V_2_CT_x_ MX was not only able to reduce the microbial burden, but also the highest concentrations of V_4_C_3_T_x_ MX exhibited an enhanced antibacterial effect against *E. coli* (Figure 4C). Consequently, a statistically significant reduction in terms of percentage of control was observed for 500 µg/mL (−16.6%) (*p* < 0.0001), 200 µg/mL (−8.8%) (*p* < 0.0001), and 100 µg/ml (−5.5%) (*p* < 0.05) of V_2_CT_x_ MX (Figure S2B). Despite this result, a flattening of the potential antimicrobial activity of the nanomaterials was noted after 24 h of incubation, returning to lower decreases of the CFUs (*p* > 0.05) (Figure 4C).

Under dynamic incubation conditions, a distinct antibacterial profile was observed for *S. aureus*. After 4 h of rotation, V₂CTₓ induced only marginal reductions in bacterial burden, reaching 0.07 and 0.11 average log less at 500 and 200 µg/mL, respectively (*p* > 0.05), compared to untreated bacteria. In contrast, V₄C₃Tₓ exhibited a markedly enhanced antibacterial effect under the same conditions, with an average decrease of 2.37 and 1.40 logs at 500 and 200 µg/mL (*p* < 0.0001; *p* < 0.001) (Figure 4E). In terms of percentage variation relative to untreated controls, these reductions corresponded to −11.0% and ‐8.7% at 500 and 200 µg/ml of V₄C₃Tₓ, respectively (*p* < 0.0001), while V₂CTₓ induced percentage decreases of −16.6% and −8.8% at 500 and 200 µg/mL, respectively (*p* < 0.05 and *p* > 0.05, respectively) (Figure 2D). After 24 h of dynamic incubation, the antibacterial activity of both V₂CTₓ activity at 500 µg/mL reduced the bacterial burden by 1.14 log (*p* < 0.0001) and V₄C₃Tₓ at the same concentration resulted in an equivalent 1.14 log reduction (*p* < 0.0001). At 200 µg/mL, V₂CTₓ and V₄C₃Tₓ reduced CFU by 1.11 and 1.05 logs, respectively (*p* < 0.001).

**Figure 5:**
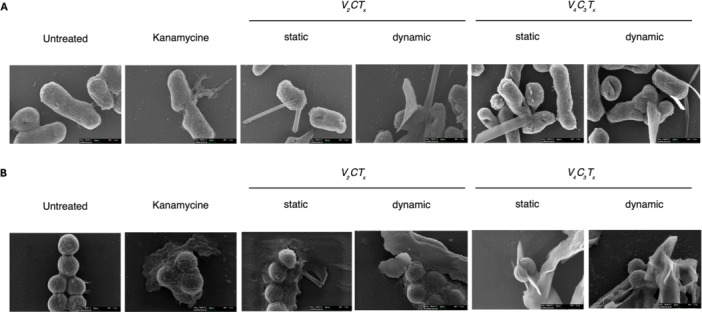
Scanning electron microscopy (SEM) analysis of bacterial morphology following MXene treatment. Representative SEM images of *E. coli* (A) and *S. aureus* (B) after 4 h of incubation (scale bar: 200 nm). Bacteria were untreated, treated with kanamycin as antibiotic control, or exposed to V₂CTₓ or V₄C₃Tₓ MXenes (500 µg/mL) under static or dynamic incubation conditions. Untreated bacteria displayed intact cell morphology, while MXene‐treated samples showed physical association between bacterial cells and MXene flakes, observing by membrane deformation and loss of cell integrity, with more marked effects under dynamic conditions.

To assess the *“nano‐knives”* effect SEM was carried out on 4 h post‐treated specimens (Figure 5). Both MXenes V₂CTₓ and V₄C₃Tₓ, similarly in static and dynamic conditions, showed the *“nano‐knives”* interaction with bacterial cells elucidating the mechanic antibacterial activity in respect with controls. Of note, our experimental setting allowed to capture the few bacterial cells treated with the kanamycin, that does not impact directly on the cell wall of *S. aureus* or cell membrane of *E. coli*, but it impairs the protein synthesis binding irreversibly to the 30S ribosomal subunit (Cushnie et al. 2016; Krause et al. 2016), well emphasizing the mechanic stress related to MXenes' treatment.

### 3.4 Biocompatibility Assessment of V_2_CT_x_ and V_4_C_3_T_x_ Mxenes

**Figure 6:**
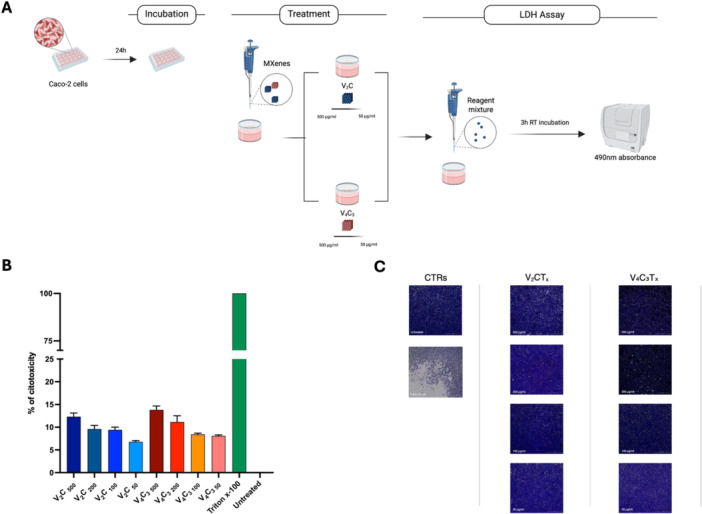
V₂CTx and V₄C₃Tx MXenes exhibit no cytotoxicity on human epithelial cells. MXenes cytotoxicity was assessed on human colorectal adenocarcinoma cell line (Caco‐2) by using LDH assay (A). Caco‐2 cell monolayer was treated with 500, 200, 100 and 50 µg/mL of V₂CTx and V₄C₃Tx MXenes. Untreated cells and cells treated with 2% Triton X‐100 were used as negative and positive controls, respectively. Cytotoxicity was determined by measuring absorbance at 490 nm wavelength using Cytation instrument following LDH assay. Measures were shown as percentage of cytotoxicity, expressed as mean ± SD and statistical significance assessed by one‐way ANOVA. (B). Cell morphology and monolayer integrity following MXene exposure were further evaluated by Crystal Violet staining (C). Images acquired with the Cytation 5 show Caco‐2 cells after treatment with V₂CTx and V₄C₃Tx at the indicated concentrations, compared with untreated controls and Triton X‐100–treated cells, confirming the absence of detectable cytotoxic or detachment effects induced by MXenes.

The toxicity of both V_2_CT_x_ and V_4_C_3_T_x_ MXs was investigated as schematically depicted in the experimental scheme (Figure 5A). The biocompatibility of MXenes was evaluated by incubating Caco‐2 eukaryotic cells with V_2_CT_x_ and V_4_C_3_T_x_ MX at final concentrations of 500, 200, 100 and 50 µg/mL, respectively. After 24 h of incubation, we observed no significant variations in terms of LDH release those accounted comparable levels between V_2_CT_x_ and V_4_C_3_T_x_ compounds (Figure 6B). To further evaluate potential delayed cytotoxic effects, extended exposure experiments up to 5 days were performed, revealing a concentration‐dependent reduction in cell viability at longer time points (Figure S3).

Although the cytoxicity of V_2_CTx MX increased based on dosage levels, it remained between 7% and 13%. Similarly, V_4_C_3_T_x_ MX showed a cytotoxic effect which ranged between 8% and 14%. To further support the LDH‐based biocompatibility data, Caco‐2 cell monolayer integrity was qualitatively assessed by CV staining (Figure 6C). Microscopic images acquired using the Cytation 5 system (Agilent Technologies, USA) showed preserved epithelial monolayer morphology after treatment with V₂CTₓ and V₄C₃Tₓ at all tested concentrations, comparable to untreated control cells. In contrast, cells treated with Triton X‐100 showed extensive monolayer disruption and cell detachment, suggesting that MXene exposure does not induce detectable cytotoxic or detachment effects on epithelial cells under the experimental conditions tested.

**Figure 7:**
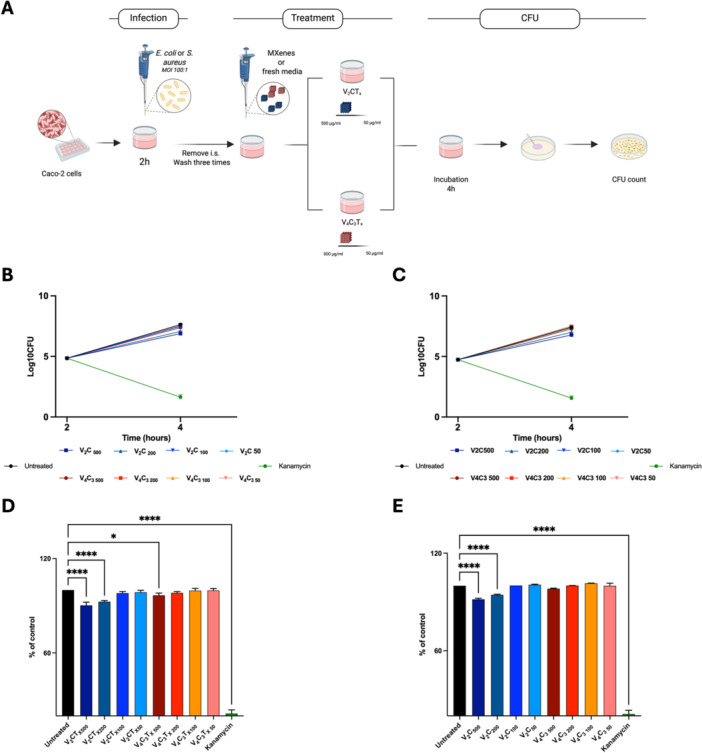
V₂CTx and V₄C₃Tx MXenes decrease *E. coli* or *S. aureus* persistence in epithelial cells. Schematic representation of the experimental model used to assess MXenes antibacterial activity on human colorectal adenocarcinoma (Caco‐2) cells or murine macrophage (J774) (A). Caco‐2 were infected with *E. coli* (B) or *S. aureus* (C) (MOI = 100:1) for 2 h before removing the infection solution and treating with 500, 200, 100 and 50 µg/mL V₂CTx and V₄C₃Tx MXenes. Colonies Forming Units (CFUs) were evaluated at 4‐ and 24‐ hours. Intracellular CFUs were reported in log10 scale as line plots. The ability of V₂CTx and V₄C₃Tx to reduce microbial burden was evaluated by quantifying bacterial growth as log10 intracellular CFU through CFU counts at 4 h in Caco‐2 cells for both *E. coli* (D) and *S. aureus* (E). Data were expressed as mean ± SD and statistical significance assessed by one‐way ANOVA.

To investigate the potential antimicrobial properties of V_2_CT_x_ and V_4_C_3_T_x_ MX, epithelial (Caco‐2) and macrophage (J774) cell lines were infected with *E. coli* (MOI 100:1) and treated with V_2_CT_x_ and V_4_C_3_T_x_ MX at final concentrations of 500, 200, 100 and 50 µg/mL (Figure 7A; Figure S3) (Rosato et al. 2024). CFUs counting was performed 4 h after the respective treatment.

Consistent with the initial observations, MXene treatments moderately attenuated the intracellular bacterial burden within infected Caco‐2 cells relative to untreated controls, exhibiting a clear dose‐dependent inhibitory profile (Figure 7B). Notably, V₂CT_x_ emerged as the more efficient MX, producing reductions of approximately 0.8 and 0.6 log units at concentrations of 500 and 200 μg/mL, respectively. Conversely, the antibacterial efficacy of V₄C₃T_x_ was markedly lower, yielding a modest 0.3 log reduction even at the maximum dosage (*p* < 0.001), while lower concentrations (100–50 μg/mL) produced negligible impact ( ~ 0.2 log units).

To evaluate whether these effects were cell‐type specific, the analysis was extended to J774 macrophages (Figure S4). Under identical experimental setting, the highest concentration (500 μg/mL) of V₂CTx induced only a marginal decrease in CFU counts ( ~ 0.3 log units; *p* > 0.05), with no statistically significant variations observed across the remaining cohorts. *E. coli* survival was additionally reported as percentage of control at the 4‐h time point (Figure 7D).

We observed a similar trend in *S. aureus*‐infected Caco‐2 cells (Figure 7C). While both MXenes reduced the intracellular bacterial burden, their efficacy varied significantly. V₂CTx proved to be more effective, achieving at 500 μg/mL a ~ 0.6 log reduction in CFU counts, with a notable effect (0.4 log) persisting at 200 μg/mL. Conversely, V₄C₃Tx showed minimal activity, yielding only a 0.1 log decrease even at the highest concentration. Concentrations under 200 μg/mL did not produce any significant impact on bacterial survival for either material. *S. aureus* burden was additionally reported as percentage of control at the 4‐h time point (Figure 7E).

### 3.5 Vanadium‐Based MXenes Regulate Reactive Oxygen Species Production in Infected Epithelial Cells

**Figure 8:**
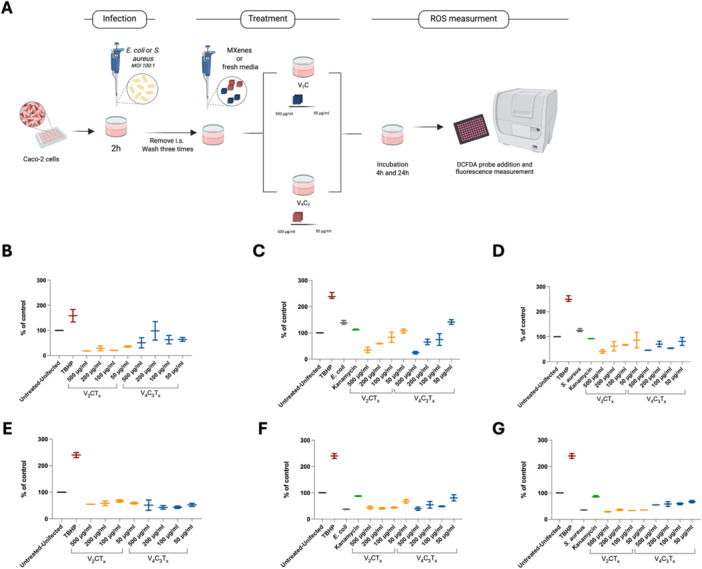
V₂CTx and V₄C₃Tx MXenes modulate intracellular ROS production in infected epithelial cells. Schematic representation of the experimental workflow used to evaluate intracellular reactive oxygen species (ROS) levels in Caco‐2 cells following bacterial infection and MXene treatment (A). Caco‐2 cells were treated with V₂CTx and V₄C₃Tx alone (B, E) or infected with *E. coli* (C, F) or *S. aureus* (D, G) at a multiplicity of infection (MOI) of 100 for 2 h. After removal of the infection inoculum and three washing steps, cells were treated with V₂CTx and V₄C₃Tx MXenes at final concentrations ranging from 500 to 50 µg/mL or maintained in fresh culture medium. Intracellular ROS levels were quantified at 4 h (B–D) and 24 h (E–G) post‐treatment using the DCFDA fluorescent probe and measured by a fluorescence plate reader.

Intracellular ROS levels were quantified to determine whether vanadium‐based MXenes exhibited oxidative stress on host epithelial cells under the same conditions used for antibacterial activity testing (Figure 8). In uninfected Caco‐2 cells, exposure to V₂CTx or V₄C₃Tx at all tested concentrations did not increase ROS at either 4 or 24 h; on the opposite, ROS levels remained consistently below basal control values (Figure 8B, E), ruling out intrinsic pro‐oxidant activity under these conditions. As a positive control, TBHP raised ROS intracellular production, confirming the success of the assay and the responsivity of the cells.

Bacterial infection triggered a substantial oxidative response in epithelial cells. At 4 h post‐infection, both *E. coli* and *S. aureus* elevated intracellular ROS to approximately 120%–150% relative to uninfected controls (Figure 8C,D). V₂CTx treatment markedly attenuated this response, reducing ROS to ~25%–50% at the highest concentrations tested, a decrease exceeding 60% compared to infected, untreated cells. V₄C₃Tx also reduced ROS accumulation, though less effectively, with residual values intermediate between infected controls and V₂CTx‐treated cells. Kanamycin‐treated infected cells retained elevated ROS levels comparable to those of untreated infected cells, indicating that ROS attenuation by MXenes was not merely secondary to bacterial reduction.

At 24 h post‐infection, ROS levels in infected cells declined partially relative to the 4 h time point but remained above basal levels (Figure 8F,G). V₂CTx‐treated cells continued to display significantly lower ROS than infected controls (*p* < 0.05), while V₄C₃Tx maintained a moderate but consistent attenuation. These results suggested that vanadium‐based MXenes have a scavenger activity.

## 4 Discussion

Despite the numerous attempts to reduce unnecessary antibiotic prescribing, aiming at decreasing selection pressure for resistance, new resistances continue to emerge and to spread. In this scenario, there is an urgent need for new antibiotics, particularly those directed against both community‐acquired or nosocomial‐pathogens and particularly multi‐drug resistant bacteria (Livermore 2004). Nanomaterial‐based antimicrobial treatments appear as a promising option against bacteria and fungi, which cannot only be used to functionalize medical devices or personal protective equipment, but also when directly used against the mentioned pathogens (De Maio 2019a; Palmieri et al. 2021; De Maio et al. 2022). Unfortunately, the lack of standardization to assess their activity and efficacy leads to opposite results for their application slowing down their likely medical applications (De Maio 2019a).

MXenes are recognized as innovative nanomaterials in the medical field for their antimicrobial properties (Seidi et al. 2023; Ferrara et al. 2024). These mechanisms can be summarized as a) the disruption of the physical membrane through sharp edges, b) the induction of photothermal effects, which directly contributes to antibacterial and antibiofilm activity, and c) the intracellular disruption inducing ROS production. Importantly, studies on antimicrobial activities of MXenes are still elusive and in their early stages, suggesting the need for certain insights especially for the application on the human host.

In this contribution, we evaluated the antimicrobial properties and biocompatibility of two vanadium‐based MXenes, V₂CT_x_ and V₄C₃T_x_, using both axenic cultures and cell infection model with *E. coli* or *S. aureus* as indicator strains. Consequently, we assessed how the structural stability of V₄C₃T_x_ compared to the higher flexibility and dispersibility of V₂CT_x_ may impair their biological activity against our indicator strain. In this regard, V₂CT_x_ exhibited a lamellar structure with thin, irregularly stacked nanosheets and folded edges, while V₄C₃T_x_ verified thicker and more compact sheets, reflecting its higher structural complexity. These structural differences may influence the interaction between MXene nanosheets and bacterial surfaces, potentially affecting their antimicrobial activity.

Vanadium‐based MXenes exhibited an antibacterial activity in a concentration‐ and condition‐dependent manner against both *E. coli* and *S. aureus*. Although the large surface area of MXene nanosheets favors close interactions with microbial membranes, our observations suggest that this factor alone is insufficient to fully account for the antibacterial activity observed. In other words, CFUs reducing was due to nano knife activity rather than a trapping effect, as highlighted by SEM analysis. Additionally, while oxidative stress is commonly invoked as a key mechanism underlying nanomaterial‐mediated antibacterial activity (Lemire et al. 2013; Hajipour et al. 2012), our findings pointed out a different effect. Both V₂CTx and V₄C₃Tx exhibited ROS‐scavenging rather than ROS‐inducing behavior, in line with previous reports describing the antioxidant and ROS‐modulating properties of the vanadium‐based MXenes (Feng et al. 2021). This finding argues against MXenes oxidative stress dependent‐ antimicrobial properties, prompting instead a major involvement of physicochemical interactions.

The use of dynamic incubation slightly enhanced the antibacterial effect, likely by promoting a closer contact between the nanosheets and bacterial membranes, thus boosting the mechanical disruption based on the physical “nano‐knife” damage (Seidi et al. 2023). This observation reinforces the idea that the intrinsic mechanical properties of MXenes play a key role in their antimicrobial activity, consistent with findings for other two‐dimensional nanomaterials such as graphene oxide (De Maio 2019b; Palmieri et al. 2018).

Furthermore, the antibacterial activity remained modest and failed to exhibit a cumulative effect over the 24‐h observation period. This suggests that MXenes, even at elevated concentrations, are insufficient to suppress rapid bacterial proliferation under the specified experimental conditions. In this context, 4‐ and 24 h represented suitable time points to evaluate early steps of the infection and bacterial persistence, especially with rapid growth bacteria. Variations in susceptibility across the tested species may be attributed to fundamental differences in cell envelope architecture and surface charge density (Wang et al. 2025). These physicochemical properties dictate the electrostatic affinity and subsequent physical interaction between the MXene nanosheets and the bacterial lipid bilayer, ultimately governing the efficacy of membrane disruption. Interestingly, this effect was opposite to what previously observed following 48 h of incubation (Seidi et al. 2023). Polyhedral oligomeric silsesquioxane functionalization notably increased the antibacterial activity against both *E. coli* and *S. aureus* (Akinay et al. 2025). This aspect also raises concerns about potential methodological biases, as the evaluation of MXenes outside of appropriate time windows or culture conditions may result in artificially increased antimicrobial potential. This implies that the wrong aspects could mask the true dynamics of bacterial survival. These misinterpretations do not only interfere with the reproducibility of the experimental protocol, but could also potentially generate sublethal exposure conditions, which could be one of the causes of the emergence of resistant phenotypes, especially in case of simultaneous antibiotic pressure.

In this context, our experimental tests attempted to fill some of the existing gaps by providing a direct comparison of two vanadium‐based MXenes with different structural characteristics (V₂CT_x_ vs. V₄C₃T_x_) against *E. coli*. Indeed, our study highlights the need to report not only the concentration and exposure times, but also a precise structural characterization of MXenes, when evaluating their biological properties. This can help to explain why we observed that V₂CT_x_ with greater dispersibility and flexibility exerted stronger antibacterial activity compared to the more compact V₄C₃T_x_. This was evident despite nearly comparable levels of cytotoxicity, likely due to the morphology of the nanosheets and the mode of interaction with microbial membranes.

These methodological limitations arise especially, when there is a strong need to develop direct comparisons of microbiological interpretation, since the results were obtained in axenic cultures for most cases and often cannot be simply translated to eukaryotic infection models. To support this claim, it was observed that, although cytotoxicity profiles in mammalian cells are often reported as acceptable (Iravani and Varma 2022; Wu et al. 2023), inter‐laboratory reproducibility remains limited.

Cytotoxicity assays revealed that both V₂CT_x_ and V₄C₃T_x_ retained a relatively safe profile, with toxicity values ranging between 7% and 14% after 24 h. On the other side, extended exposure experiments up to 5 days revealed a concentration‐dependent increase in cytotoxicity, highlighting the importance of considering exposure time when evaluating MXene biocompatibility.

These data are in line with previous reports on titanium‐based MXenes, which demonstrated a potential therapeutic window for biomedical use (Iravani and Varma 2022). In intestinal epithelial cells (Caco‐2), V₂CT_x_ greatly reduced the intracellular bacterial survival in a dose‐dependent manner, while V₄C₃T_x_ showed a milder yet detectable effect. In contrast, macrophage J774 cells were less responsive to the MX treatment, with only minimal variations in the bacterial burden. These differences may reflect the specific intracellular environments since phagocytic cells may be engulfed by MXenes internalization and may neutralize their effects more effectively than epithelial cells. These findings highlight the complexity of host‐pathogen‐nanomaterial interactions and stress as well as the importance of using appropriate models to assess their properties. In this context, Caco‐2 cells were selected because they are the most widely accepted as a surrogate of the human intestinal epithelial barrier (Ran et al. 2024; Lea 2015). Upon differentiation, Caco‐2 cells develop tight junctions, apico‐basal polarity, and transport properties that closely resemble those of normal enterocytes (Kus et al. 2023). Importantly, recent studies demonstrate that Caco‐2 cells did not exhibit altered sensitivity to nanomaterials compared to non‐tumorigenic epithelial cells, supporting their relevance for nanotoxicological and antimicrobial investigations (Voloshin et al. 2023). Furthermore, the use of immortalized and well‐characterized cell lines provides a highly controlled and reproducible experimental system. In contrast, primary or non‐immortalized epithelial cells may introduce significant biological variability due to donor‐to‐donor genetic differences, limited lifespan, and heterogeneous differentiation states, which can confound the interpretation of nanomaterial‐cell interactions, particularly in quantitative antimicrobial and cytotoxicity assays. The use of CV staining, together with LDH measure, provided a robust and physiologically relevant readout of cell viability. Indeed, CV staining is particularly suitable for these studies because of it is not affected by optical or chemical interference from nanomaterials, which is a known limitation of fluorimetric live/dead assays. Likewise, flow cytometry requires detachment of adherent cells, a procedure that may mechanically damage cells or selectively detach compromised cells, thereby artificially overestimating cytotoxicity. In this context, we did not extend the exposure for long time (up to 3–5 days) to avoid medium replacement or supplementation, which would be required to maintain cell viability over longer periods and would meaningfully perturb the microenvironment (Albanese et al. 2012; Riss TL and Niles 2013; Dusinska 2019). Such interventions could alter both MXenes stability and cellular responses, introducing confounding variables that would compromise the interpretation of cytotoxicity. Therefore, we prioritized a controlled and physiologically relevant exposure window under constant medium conditions investigated by complementary assays such as LDH quantification and CV staining.

The apparent coexistence of antibacterial activity and biocompatibility in MXenes has been reported in several studies and is generally attributed to differences in interaction mechanisms between bacterial and eukaryotic cells. In particular, the antibacterial activity is associated with physical membrane disruption (“nano‐knife” effect) and surface contact interactions rather than strong chemical toxicity, which can explain why moderate antibacterial effects may occur while maintaining acceptable cytocompatibility toward mammalian cells. No significant chlorine‐related signals were detected in the XPS spectra, suggesting that Cl‐containing species are not present in relevant concentrations and antibacterial effects are not caused by contaminants. Furthermore, the biological results do not support a toxicity mechanism dominated by residual chemical species. If antibacterial activity were primarily driven by residual ions or etching by‐products, a comparable cytotoxic effect toward eukaryotic cells would be expected.

In conclusion, our results suggest that vanadium‐based MX may be more readily exploited in technological or preventive contexts rather than direct therapeutic use, as previously hypothesized for graphene oxide (Palmieri et al. 2021). Examples include their integration into coatings for medical devices, personal protective equipment, or hospital surfaces, where a moderate antimicrobial activity combined with good biocompatibility in an unfavorable environment for bacterial growth would be advantageous. On the other hand, the ability to modulate ROS and to exploit photothermal/photodynamic effects, should require further investigation maintaining appropriate conditions and experimental models. While a detailed kinetic analysis of oxidation is beyond the scope of this work, the comparison between fresh and aged samples provides a representative framework to evaluate how surface oxidation under aqueous conditions influences the biological response.

Graphs show intracellular ROS levels expressed as percentage of the untreated–uninfected control. TBHP was used as a positive control for ROS induction, while kanamycin‐treated infected cells were included as an antibiotic control. Data are reported as mean ± SD. Statistical significance was assessed by one‐way ANOVA.

## Author Contributions

**Roberto Rosato:** investigation, writing – original draft, methodology, writing – review and editing, formal analysis, data curation. **Andreas Rosenkranz:** conceptualization, investigation, writing – original draft, methodology, validation, visualization, writing – review and editing, formal analysis. **Giordano Perini:** investigation, methodology, formal analysis, writing – review and editing. **Giulia Santarelli:** investigation, visualization, formal analysis, data curation, writing – review and editing. **Alberto Augello:** methodology, investigation. **Dario F. Zambrano:** conceptualization, investigation, writing – review and editing, data curation. **Iasi Cervantes:** conceptualization, investigation, writing – review and editing. **Nuria Abigail Plebani:** conceptualization, investigation, writing – review and editing, data curation. **Fernando Pablo Cometto:** conceptualization, investigation, validation, supervision. **Marco De Spirito:** conceptualization, investigation, visualisation, writing – review and editing. **Maurizio Sanguinetti:** conceptualization, investigation, funding acquisition, writing – review and editing, visualization, software, data curation, resources, formal analysis. **Valentina Palmieri:** conceptualization, investigation, writing – review and editing, software, formal analysi, supervision. **Massimiliano Papi:** conceptualization, investigation, funding acquisition, writing – original draft, methodology, visualization, formal analysis. **Flavio De Maio:** conceptualization, investigation, writing – original draft, methodology, validation, visualization, writing – review and editing, software, formal analysis, project administration, data curation, supervision, resources.

## Ethics Statement

The authors have nothing to report.

## Consent

The authors have nothing to report.

## Conflicts of Interest

The authors declare no conflicts of interest.

## Supporting information


**Figure S1:** Valence Band spectrum at the Fermi edge obtained by XPS for the V₄C sample (A); first derivative of the Valence Band. It can be observed that the correction applied to the spectra acquired for this surface is 0.35 eV (B).

**Figure S2:** Bacterial survival at the 4‐hour time point, expressed as percentage of control, *in E. coli*‐infected cells under static (A) and dynamic (B) conditions and in S*. aureus*‐infected cells under static (**C**) and dynamic (D) conditions.

**Figure S3:** Mid‐term cytotoxicity assessment of V₂CTₓ and V₄C₃Tₓ up to 5 days. MXenes cytotoxicity was evaluated on human colorectal adenocarcinoma cell line (Caco‐2). Cells were treated with V₂CTₓ and V₄C₃Tₓ at final concentrations of 500, 200, 100 and 50 µg/mL and incubated for up to 5 days (120 hours) under standard culture conditions. Cell monolayer integrity and viability were assessed by Crystal Violet (CV) staining. Representative images acquired with the Cytation 5 system show Caco‐2 cells after treatment with V₂CTₓ and V₄C₃Tₓ at the indicated concentrations, compared with untreated controls and Triton X‐100–treated cells.

**Figure S4:** Schematic representation of the experimental workflow used to evaluate the antibacterial activity of MXenes in murine macrophages (J774) (A). Cells were infected with *E. coli* (B) or *S. aureus* (C) (MOI = 100:1) for 2 h, after which the infection medium was removed, and cells were washed three times to eliminate extracellular bacteria. Cells were then treated with V₂CTx or V₄C₃Tx MXenes at 500, 200, 100, or 50 μg/mL and incubated for 4 h before bacterial quantification by colony‐forming unit (CFU) counting. Quantification of bacterial survival at the 4‐hour time point in *E. coli* (D) or *S. aureus*‐infected (E) J774 macrophages, expressed as percentage of control. Data are presented as mean ± SD. Statistical significance was assessed by one‐way ANOVA.

## Data Availability

Data may be available upon request to the corresponding author. The data that support the findings of this study are available on request from the corresponding author. The data are not publicly available due to privacy or ethical restrictions.
