# Supplementary material for: Exploring the Antimicrobial Potential of Vanadium‐Based MXenes for Biomedical Applications
Source: Microbiologyopen. 2026 May 18;15(3):e70309. doi: 10.1002/mbo3.70309 (PMC13181600; doi:10.1002/mbo3.70309)
Supplement: Supplementary file 1 — Figure S1: Valence Band spectrum at the Fermi edge obtained by XPS for the V₄C sample (A); first derivative of the Valence Band. It can be observed that the correction applied to the spectra acquired for this surface is 0.35 eV (B). [file MBO3-15-e70309-s004.docx]

**Supplementary data 1**

The binding energy (BE) calibration was carried out following the procedure proposed by Näslund et al.¹ According to this method, each high-resolution spectrum is corrected by an amount equal to the shift observed in the Fermi edge (Figure S1a), measured from the valence band spectrum, with respect to the reference value of BE = 0 eV. To determine this shift, the first derivative of the valence band spectrum around the Fermi edge is calculated (gray curve in Figure S1b). Figure S1 displays both the valence band region near the Fermi edge and its corresponding derivative. As shown, a shift of approximately 0.35 eV relative to BE = 0 eV is observed; consequently, this correction is applied to both the valence band and the corresponding core-level spectra (C 1s, O 1s, V 2p, and F 1s). This procedure was repeated for each analyzed sample and surface.


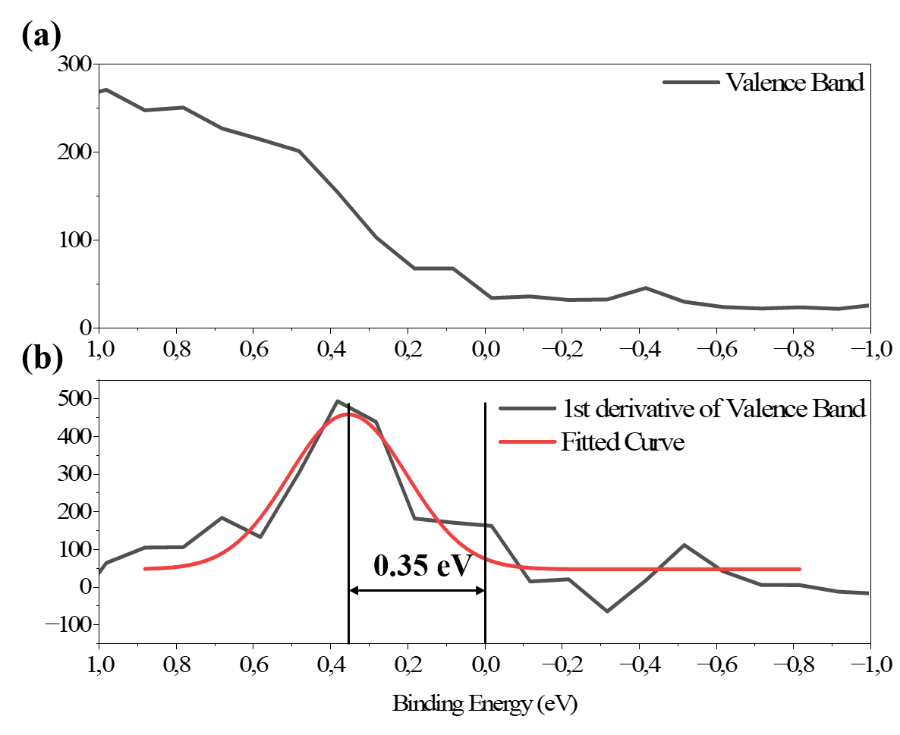


**Figure S1.** (a) Valence Band spectrum at the Fermi edge obtained by XPS for the V₄C sample; (b) first derivative of the Valence Band. It can be observed that the correction applied to the spectra acquired for this surface is 0.35 eV.
