# Supplementary material for: Exploring the Antimicrobial Potential of Vanadium‐Based MXenes for Biomedical Applications
Source: Microbiologyopen. 2026 May 18;15(3):e70309. doi: 10.1002/mbo3.70309 (PMC13181600; doi:10.1002/mbo3.70309)
Supplement: Supplementary file 2 — Figure S2: Bacterial survival at the 4‐hour time point, expressed as percentage of control, in E. coli‐infected cells under static (A) and dynamic (B) conditions and in S. aureus‐infected cells under static (C) and dynamic (D) conditions. [file MBO3-15-e70309-s003.docx]

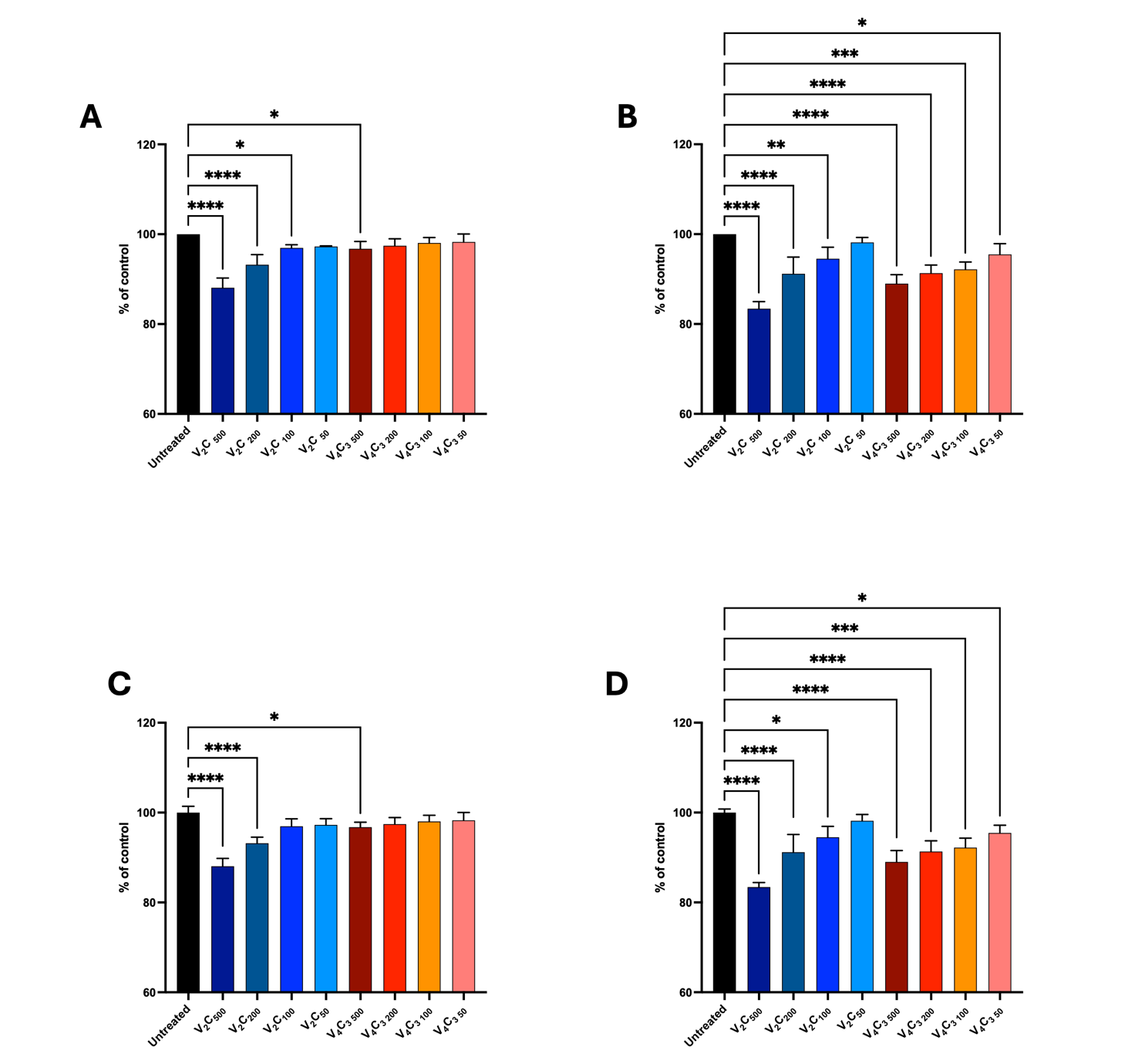


**Figure S2.** Bacterial survival at the 4-hour time point, expressed as percentage of control, *in E. coli*-infected cells under static (**A**) and dynamic (**B**) conditions and in S*. aureus*-infected cells under static (**C**) and dynamic (**D**) conditions.
