# Supplementary figures and images for: Exploring the Antimicrobial Potential of Vanadium‐Based MXenes for Biomedical Applications
Source: Microbiologyopen. 2026 May 18;15(3):e70309. doi: 10.1002/mbo3.70309 (PMC13181600; doi:10.1002/mbo3.70309)

**Supplementary data 3. Mid-term cytotoxicity assessment of V₂CTₓ and V₄C₃Tₓ up to 5 days**

**
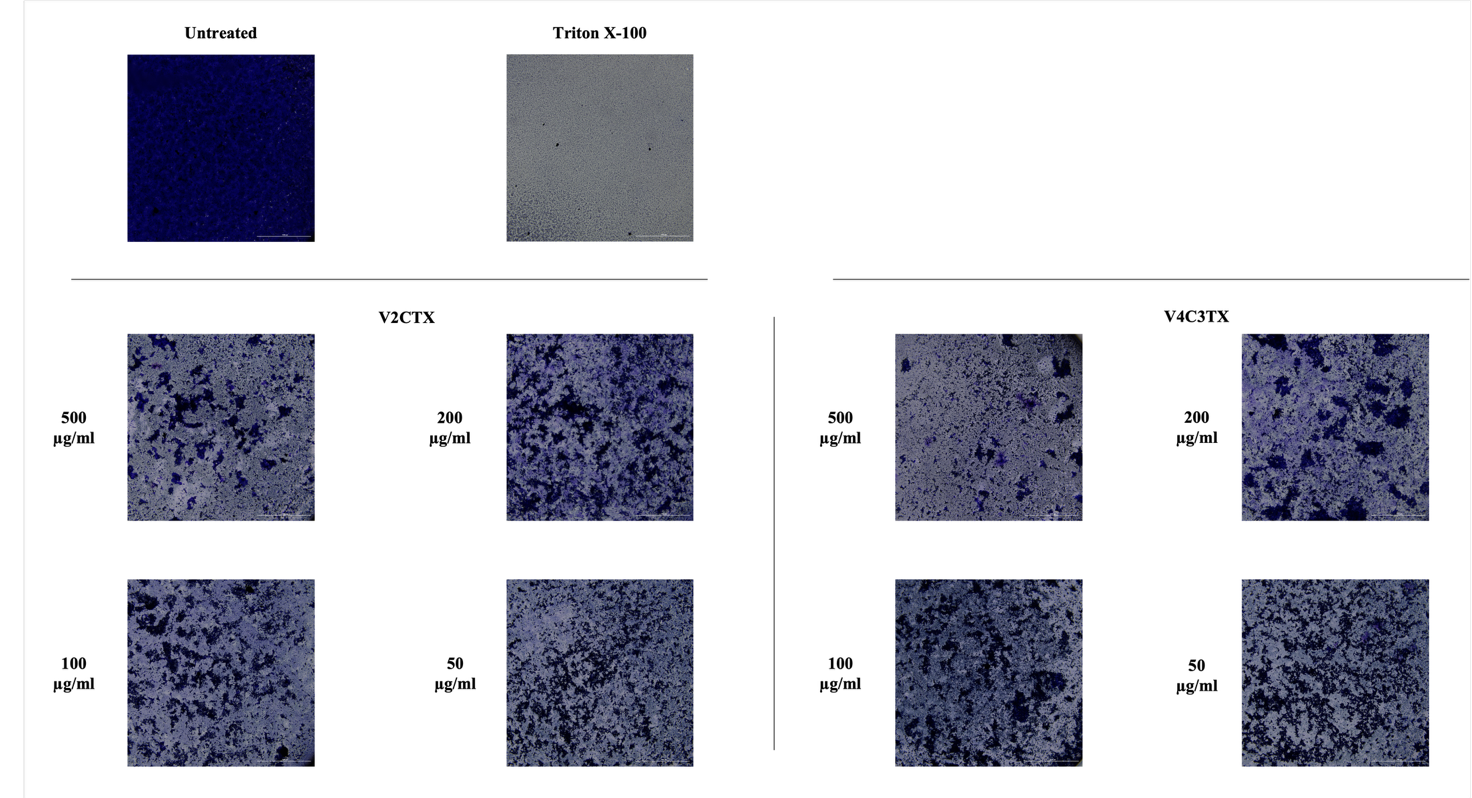
**

Supplement: Supplementary file 3 — Figure S3: Mid‐term cytotoxicity assessment of V₂CTₓ and V₄C₃Tₓ up to 5 days. MXenes cytotoxicity was evaluated on human colorectal adenocarcinoma cell line (Caco‐2). Cells were treated with V₂CTₓ and V₄C₃Tₓ at final concentrations of 500, 200, 100 and 50 µg/mL and incubated for up to 5 days (120 hours) under standard culture conditions. Cell monolayer integrity and viability were assessed by Crystal Violet (CV) staining. Representative images acquired with the Cytation 5 system show Caco‐2 cells after treatment with V₂CTₓ and V₄C₃Tₓ at the indicated concentrations, compared with untreated controls and Triton X‐100–treated cells. [file MBO3-15-e70309-s002.docx]
