# Supplementary material for: Exploring the Antimicrobial Potential of Vanadium‐Based MXenes for Biomedical Applications
Source: Microbiologyopen. 2026 May 18;15(3):e70309. doi: 10.1002/mbo3.70309 (PMC13181600; doi:10.1002/mbo3.70309)
Supplement: Supplementary file 4 — Figure S4: Schematic representation of the experimental workflow used to evaluate the antibacterial activity of MXenes in murine macrophages (J774) (A). Cells were infected with E. coli (B) or S. aureus (C) (MOI = 100:1) for 2 h, after which the infection medium was removed, and cells were washed three times to eliminate extracellular bacteria. Cells were then treated with V₂CTx or V₄C₃Tx MXenes at 500, 200, 100, or 50 μg/mL and incubated for 4 h before bacterial quantification by colony‐forming unit (CFU) counting. Quantification of bacterial survival at the 4‐hour time point in E. coli (D) or S. aureus‐infected (E) J774 macrophages, expressed as percentage of control. Data are presented as mean ± SD. Statistical significance was assessed by one‐way ANOVA. [file MBO3-15-e70309-s001.docx]

**Macrophage Cells Culture and Infection**

Murine macrophages (J774 A.1 cell line) were cultured in Dulbecco’s Modified Eagle’s Medium (DMEM) (Euroclone, Milano, Italy) supplemented with 10% inactivated fetal bovine serum (FBS) (Euroclone, Italy), 1% L-glutamine, and 1% streptomycin–penicillin (Euroclone). Cells were maintained at 37 °C in a humidified atmosphere containing 5% CO₂. Adherent cells were washed with sterile warm phosphate-buffered saline (PBS) (Euroclone) and detached using 1× trypsin in PBS (Euroclone) to be used in subsequent experiments. Cells were counted and re-suspended in DMEM supplemented with 2% FBS and 1% L-glutamine. Subsequently, cells were seeded in sterile 48-well plates (Euroclone) at a concentration of 1.2 × 10⁶ cells/mL and incubated overnight to allow adherence prior to infection or treatment.

J774 cells were infected with *E. coli* or *S. aureus* at a multiplicity of infection (MOI) of 100 (100 bacteria per cell), with bacteria resuspended in the cell culture medium. Two-hour post-infection, cells were gently washed with sterile warm PBS to remove extracellular bacteria and treated with either V_2_CT_x_ and V_4_C_3_T_x_ MX at final concentrations of 500, 200, 100 and 50 µg/mL, respectively. Following treatment, cells were incubated for 4 and 24 hours under standard conditions.

To determine the intracellular bacterial survival, the monolayer was harvested using 0.1 mL of sterile 0.05% Triton X-100 (Sigma-Aldrich, USA). Serial 1:10 dilutions were performed in PBS and plated on LB agar plates. Plates were incubated overnight at 37 °C. CFUs/mL were calculated by multiplying the number of colonies by the corresponding dilution factor and reported as log CFUs/10^6^ cells. The schematic flowchart is reported in results.


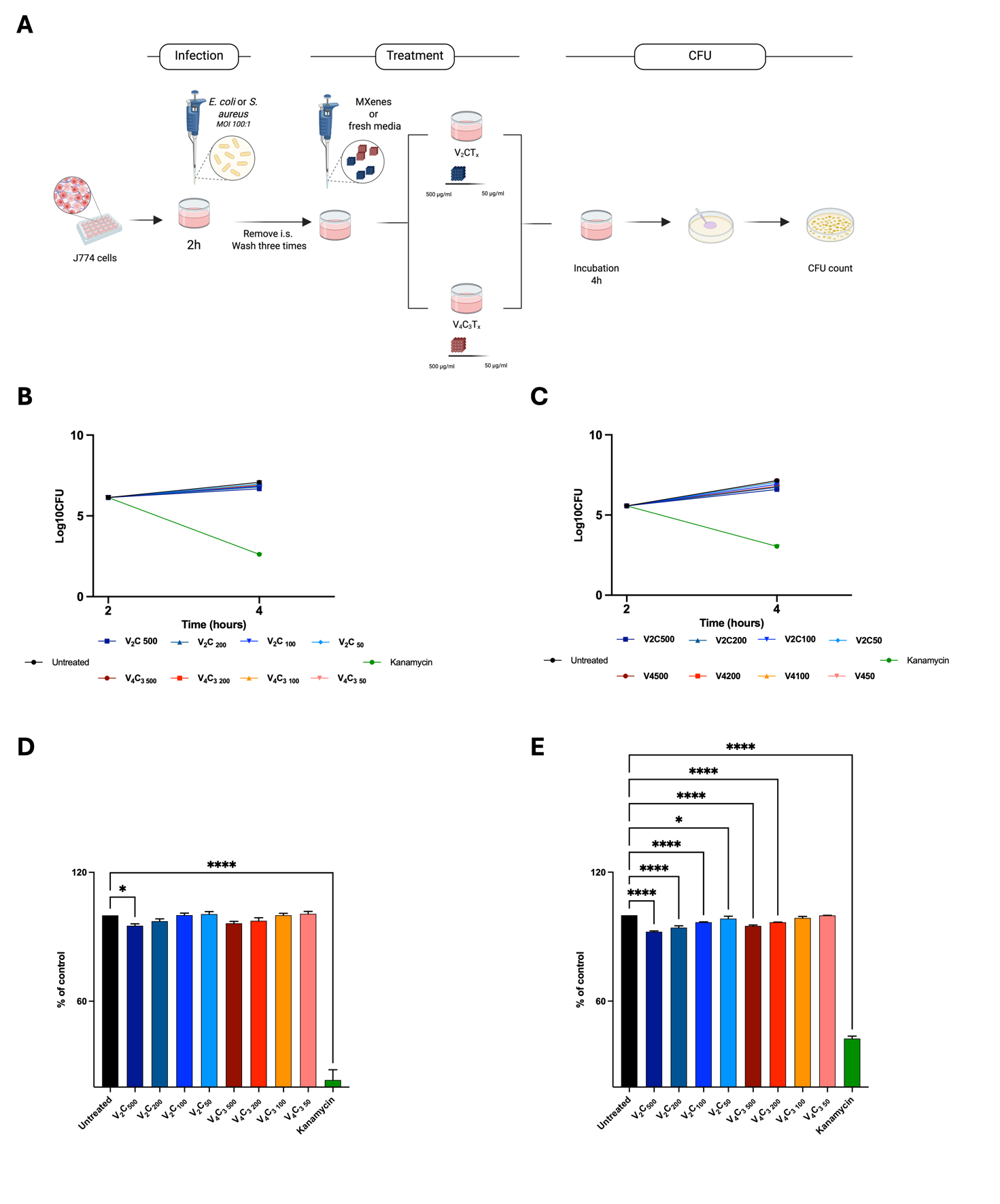


**Figure S3.** Schematic representation of the experimental workflow used to evaluate the antibacterial activity of MXenes in murine macrophages (J774) (**A**). Cells were infected with *E. coli* (**B**) or *S. aureus* (**C**) (MOI = 100:1) for 2 h, after which the infection medium was removed, and cells were washed three times to eliminate extracellular bacteria. Cells were then treated with V₂CTx or V₄C₃Tx MXenes at 500, 200, 100, or 50 μg/mL and incubated for 4 h before bacterial quantification by colony-forming unit (CFU) counting. Quantification of bacterial survival at the 4-hour time point in *E. coli* (**D**) or *S. aureus-*infected (**E**) J774 macrophages, expressed as percentage of control. Data are presented as mean ± SD. Statistical significance was assessed by one-way ANOVA.
